# Transcriptional profiling demonstrates altered characteristics of CD8 ^+^ cytotoxic T‐cells and regulatory T‐cells in *TP53‐*mutated acute myeloid leukemia

**DOI:** 10.1002/cam4.4661

**Published:** 2022-03-16

**Authors:** Milad Abolhalaj, Viktor Sincic, Henrik Lilljebjörn, Carl Sandén, Alar Aab, Karin Hägerbrand, Peter Ellmark, Carl A. K. Borrebaeck, Thoas Fioretos, Kristina Lundberg

**Affiliations:** ^1^ Department of Immunotechnology, Medicon Village (Building 406) Lund University Lund Sweden; ^2^ CREATE Health Cancer Center, Medicon Village (Building 406) Lund University Lund Sweden; ^3^ Division of Clinical Genetics, Department of Laboratory Medicine Lund University Lund Sweden; ^4^ Alligator Bioscience AB Medicon Village Lund Sweden; ^5^ Department of Clinical Genetics and Pathology University and Regional Laboratories Region Skåne Lund Sweden

**Keywords:** acute myeloid leukemia, immunotherapy, RNA‐sequencing, T‐cells, *TP53*

## Abstract

**Background:**

Acute myeloid leukemia (AML) patients have limited effect from T‐cell‐based therapies, such as PD‐1 and CTLA‐4 blockade. However, recent data indicate that AML patients with *TP53* mutation have higher immune infiltration and other immunomodulatory therapies could thus potentially be effective. Here, we performed the transcriptional analysis of distinct T‐cell subpopulations from *TP53*‐mutated AML to identify gene expression signatures suggestive of altered functional properties.

**Methods:**

CD8^+^ cytotoxic T lymphocytes (CTLs), conventional helper T cells (Th), and regulatory T cells (Tregs) were sorted from peripheral blood of AML patients with *TP53* mutation (n = 5) and healthy donors (*n* = 3), using FACS, and the different subpopulations were subsequently subjected to RNA‐sequencing. Differentially expressed genes were identified and gene set enrichment analysis (GSEA) was performed to outline altered pathways and exhaustion status. Also, expression levels for a set of genes encoding established and emerging immuno‐oncological targets were defined.

**Results:**

The results showed altered transcriptional profiles for each of the T‐cell subpopulations from *TP53*‐mutated AML as compared to control subjects. IFN‐α and IFN‐γ signaling were stronger in *TP53*‐mutated AML for both CTLs and Tregs. Furthermore, in *TP53*‐mutated AML as compared to healthy controls, Tregs showed gene expression signatures suggestive of metabolic adaptation to their environment, whereas CTLs exhibited features of exhaustion/dysfunction with a stronger expression of *TIM3* as well as enrichment of a gene set related to exhaustion.

**Conclusions:**

The results provide insights on mechanisms underlying the inadequate immune response to leukemic cells in *TP53*‐mutated AML and open up for further exploration toward novel treatment regimens for these patients.

## Graphical abstract

Cytotoxic and regulatory T‐cells are transcriptionally altered in AML patients with TP53 mutation. The transcriptional profiles suggest metabolic adaptation and proliferation of Tregs, and exhaustion/dysfunction of cytotoxic T‐cells. Hence, these mechanisms could contribute to the inadequate immune response to leukemic cells and thus pave the way for novel treatment regimens for these patients.

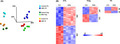

## 1 INTRODUCTION

Acute myeloid leukemia (AML) is the most common acute leukemia in adults.^1^ It involves the transformation of hematopoietic stem or progenitor cells, whose progeny expands to outcompete normal bone marrow cells and reduces the levels of functional red/white blood cells and platelets.^2^ Chemotherapy with or without transplantation has remained the first line of therapy for decades, however, relapse is very common.^2^ The 5‐year survival rate is only 5%–15% for patients older than 60 years, and around 40% for patients below the age of 60.^2^

T‐cell modulation by checkpoint inhibitors that target the PD‐1/PD‐L1 interaction, and thereby release the breaks of CD8^+^ cytotoxic T lymphocytes (CTLs), have shown unprecedented effects in several cancers.^3^ While AML‐CTLs have shown signs of dysfunction and exhaustion,^4^ the effect of PD‐1 checkpoint blockade in AML patients have thus far been limited.^5^ The reason for this is currently unclear but it could be due to low mutational burden and/or suppression of CTL response mediated by mechanisms/pathways other than PD‐1. Additionally, other immune cell populations such as myeloid cells, regulatory T‐cells (Tregs), or conventional helper T cells (Th) could be involved.^6^, ^7^, ^8^

*TP53* aberrations occur in around 13% of AML patients and are associated with treatment resistance and very poor prognosis, thus underscoring the need for new treatment regimens for this subset of patients.^9^, ^10^ Recently, gene expression profiling of bone marrow samples of *TP53*‐mutated AML has demonstrated a higher mutational burden and immune cell infiltration compared to AMLs with other risk‐defining molecular lesions.^11^ Additionally, *TP53*‐mutated AML has been shown to be strongly correlated with an IFN‐γ‐dominant microenvironment.^11^, ^12^ Altogether, this suggests that AML patients with *TP53* mutation could benefit from T‐cell modulatory therapy, and as PD‐1 therapy have shown limited effect in AML, other targets could potentially be better suited.^5^

In this study, we performed transcriptional profiling of different subpopulations of T‐cells (CTLs, Tregs, and Th cells) in a cohort of patients with *TP53*‐mutated AML as well as in healthy controls. The results show transcriptional alterations suggestive of metabolic adaptation and proliferation for AML‐Tregs, whereas features of exhaustion were associated with AML‐CTLs. Furthermore, the gene encoding the exhaustion marker and emerging drug target *TIM3* was expressed at higher levels by AML‐CTLs than in CTLs from healthy controls.

## 2 MATERIALS AND METHODS

### 2.1 Patients and sample preparation

Peripheral blood mononuclear cells (PBMCs) from untreated AML patients with *TP53* mutation (*n* = 5, see Table S1 for patient characteristics) were collected and cryopreserved as part of normal diagnostic procedures at the Department of Clinical Genetics, Lund, Sweden. Control PBMCs were collected from anonymized healthy volunteers (*n* = 3) at the local blood bank (Skåne University Hospital). The research was performed in accordance with relevant guidelines/regulations and was approved by the Regional Ethics Committee (EPN‐Regionala Etikprövningsnämnden i Lund) and informed consent was obtained from all AML patients.

### 2.2 Sorting and RNA extraction

**FIGURE 1:**
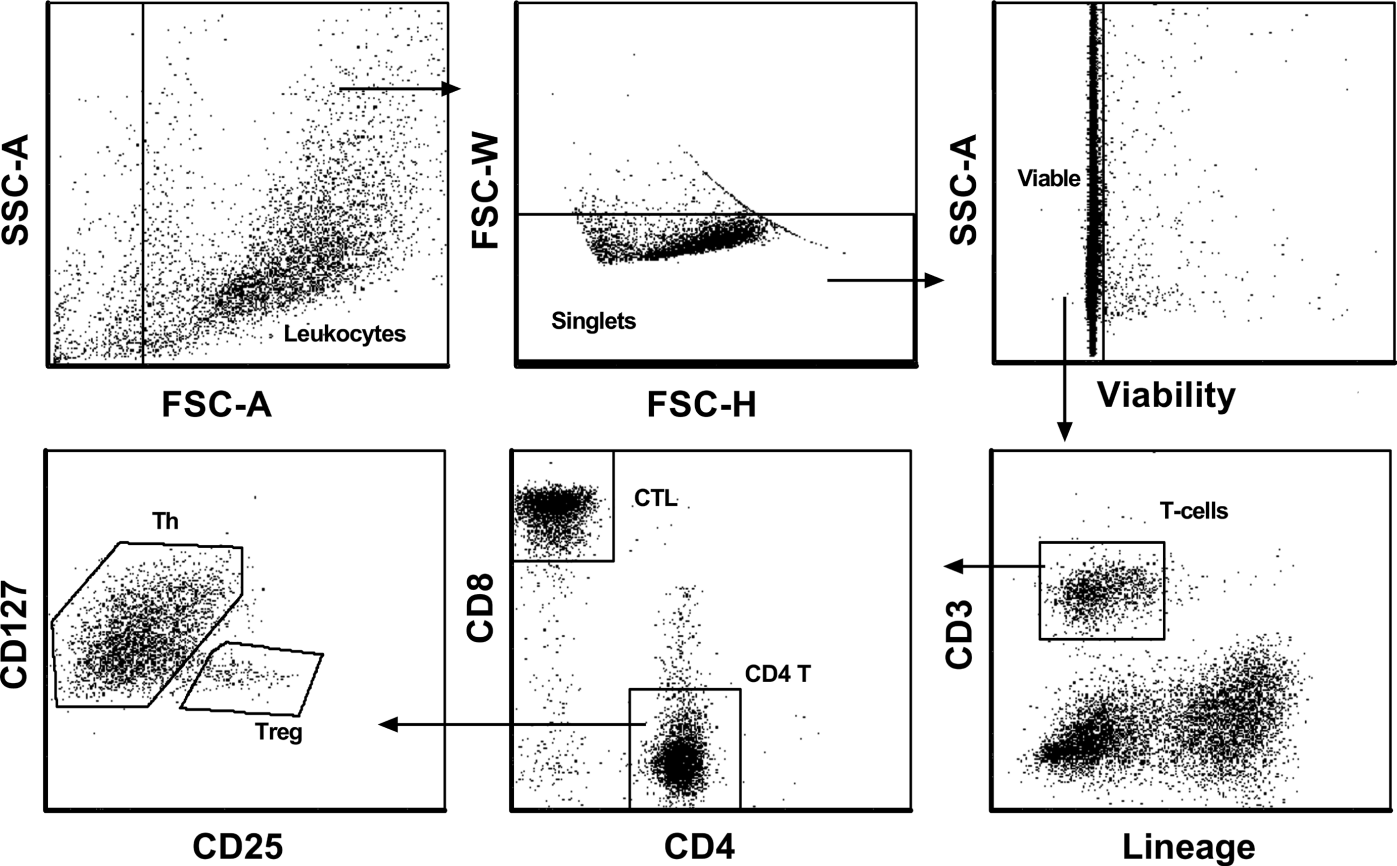
Gating strategy used for sorting CTLs, Tregs, and Th cells from peripheral blood of AML patients and control subjects. Viable leukocytes were gated out of singlets. Then, CTLs and Tregs were identified as CD3^+^Lin^−^CD4^−^CD8^+^ and CD3^+^Lin^−^CD4^+^CD25^+^CD127^−/dim^, respectively, and finally Th cells as remaining CD4^+^ T‐cells. AML, acute myeloid leukemia; CTL, cytotoxic T‐cells; Th, conventional helper T‐cells; Treg, regulatory T‐cells

Thawed cells were stained with fixable viability stain 620 (BD Biosciences) according to the manufacturer's protocol. Subsequently, cells were washed in cold PBS (GE Healthcare Biosciences, Piscataway, NJ) + 2% Fetal Bovine Serum (GIBCO), then blocked with mouse IgG (Jackson ImmunoResearch), and finally stained with an antibody panel (Table 1) for 20 min at 4 °C in Brilliant Stain Buffer (BD Biosciences). BD FACS Aria Fusion (BD Biosciences) was used for analyzing and sorting specific T‐cells (purity >95%) (Gating strategy outlined in Figure 1). Briefly, viable cells were gated out of singlets and T‐cells identified as CD3^+^lineage^−^ (Lin: CD14, CD16, CD19, CD56, CD66b) cells. Out of T‐cells, CTLs (CD3^+^CD8^+^) and CD4^+^ T‐cells were gated. Then, Tregs were further identified as CD4^+^CD25^+^CD127^−/dim^ cells and Th cells as the remaining CD4^+^ T‐cells. RNA was extracted from sorted T‐cells using Arcturus PicoPure RNA Isolation Kit (ThermoFisher Scientific, Waltham, MA) according to the manufacturer's instruction.

**TABLE 1:** Antibodies and viability stain used for sorting of the three T‐cell subpopulations from AML and control subjects

Staining agent	Clone	Supplier	Cat#	RRID
Fixable Viability Stain 620	–	BD biosciences	564996	AB_2869636
PerCP‐Cy5.5 Mouse Anti‐human CD19	HIB19	Biolegend	302230	AB_2073119
PerCP‐Cy5.5 Mouse Anti‐Human CD56	B159	BD biosciences	560842	AB_2033964
PerCP‐Cy5.5 Mouse Anti‐Human CD66b	G10F5	BD biosciences	562254	AB_11154419
PerCP‐Cy5.5 Mouse Anti‐Human CD14	MφP9	BD biosciences	562692	AB_2737726
PerCP‐Cy5.5 Mouse Anti‐Human CD16	3G8	BD biosciences	560717	AB_1727434
BV786 Mouse Anti‐Human CD3	SK7	BD biosciences	563799	AB_2744384
PE‐Cy7 Mouse Anti‐Human CD8	RPA‐T8	BD biosciences	557746	AB_396852
BV605 Mouse Anti‐Human CD4	RPA‐T4	BD biosciences	562658	AB_2744420
BV421 Mouse Anti‐Human CD127	HIL‐7R‐M21	BD biosciences	562436	AB_11151911
APC Mouse Anti‐Human CD25	M‐A251	BD biosciences	555434	AB_398598

### 2.3 RNA quality control and sequencing

RNA concentration and integrity were analyzed with the Agilent 2100 bioanalyzer (Agilent Technologies), using the RNA 6000 pico kit (Agilent Technologies). Amplified cDNA was prepared from the RNA using the SMART‐seq v4 ultra‐low input RNA kit (Takara Biosciences), and cDNA sequencing libraries were prepared using the Nextera Library DNA preparation kit (Illumina, CA). The libraries were sequenced by a NextSeq 500 (Illumina), using paired‐end 151 bp reads. The reads were aligned to the human reference genome hg19 using STAR 2.5.0a.^13^ Gene expression values were determined from the aligned data using cuffnorm 2.2.1.^14^ Counts were normalized and transformed into log2 FPKM values. Samples with more than 50% of reads mapping to the human genome and more than 2 million mapping reads in total were included in the analysis. Sample identities were cross‐validated by genotyping of expressed SNPs using bcftools gtcheck.

### 2.4 Gene expression analysis

Gene expression profiles were analyzed using Omics Explorer 3.2 (Qlucore). To identify differentially expressed genes among specific T‐cell subpopulations from both AML patients and control subjects, ANOVA was performed (False Discovery Rate [FDR] < 0.05), and principal component analysis (PCA) was used to visualize the data. Additionally, significantly differentially expressed genes between T‐cell subpopulations in AML as compared to the corresponding control subpopulation were identified using a two‐tailed t‐test with FDR <0.05.

### 2.5 Gene set enrichment analysis

Gene set enrichment analysis (GSEA) was performed to determine if a gene set of interest was statistically enriched in one condition compared to another.^15^ The analysis was performed in Qlucore using variable‐permutation and the Hallmark gene sets provided by the Broad Institute (comprising 50 gene sets associated with well‐defined biological states and processes).^16^ To evaluate exhaustion status of CTLs and Th cells in AML, GSEA was performed using gene sets comprising exhaustion signatures derived from peripheral blood CD8^+^ T‐cells in progressive HIV (GSE24081, from the Broad institute^17^) as well as from exhausted CD8^+^ T‐cells infiltrating the liver (Zheng et al^18^) and melanoma tumors (Tirosh et al^19^). Gene sets with FDR <0.05 were considered significantly enriched in the comparison made.

## 3 RESULTS

### 3.1 CTL, Treg, and Th cells from AML patients have distinct transcriptional profiles

**FIGURE 2:**
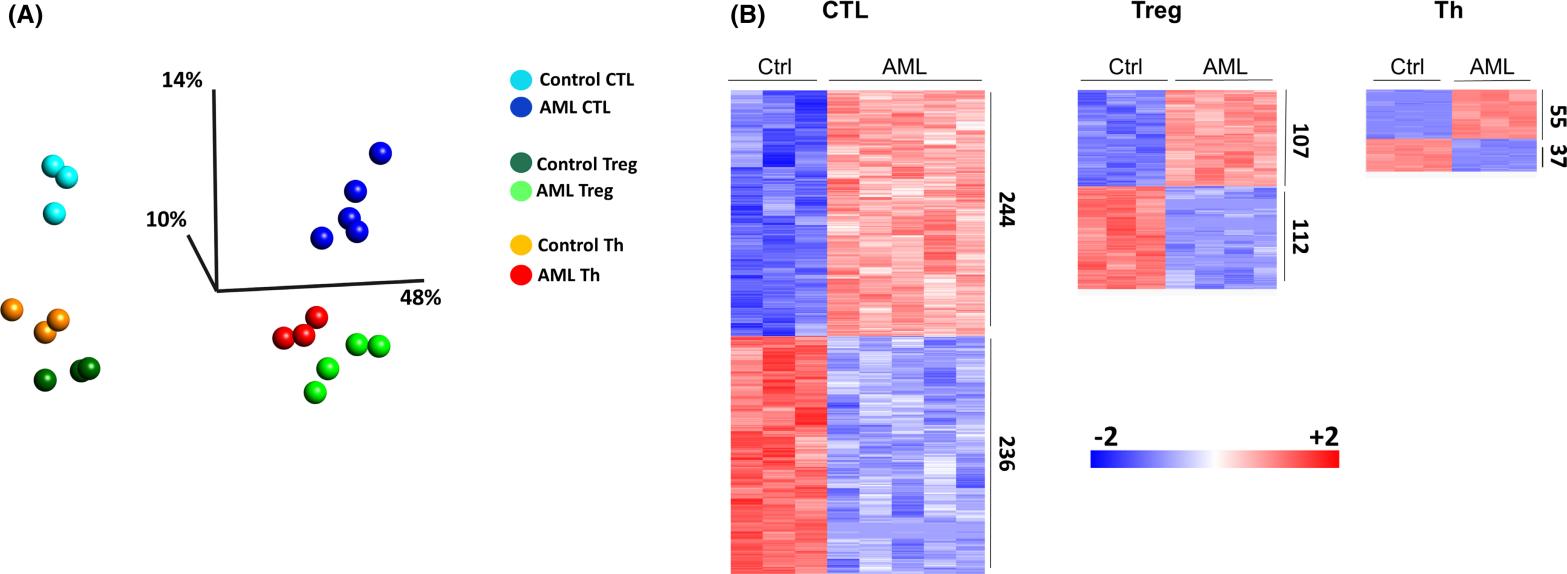
(A) PCA plot based on the 3698 differentially expressed genes among CTLs, Tregs, and Th cells from AML patients and control subjects (ANOVA, FDR < 0.05). (B) Heatmaps illustrating genes differentially expressed in CTLs, Tregs, and Th cells from AML patients as compared with the corresponding subpopulations from healthy controls (t‐test, FDR < 0.05). AML, acute myeloid leukemia; ANOVA, analysis of variance; CTL, cytotoxic T‐cells; FDR, false discovery rate; PCA, principal component analysis; Th, conventional helper T‐cells; Treg, regulatory T‐cells

To understand how T‐cells are affected in *TP53*‐mutated AML, differentially expressed genes were investigated. In total, 3698 genes were found to be differentially expressed among the six subpopulations i.e., CTLs, Tregs, and Th cells from AML and healthy control subjects. Transcriptional differences among T‐cell subpopulations from AML patients and control subjects were visualized in a PCA plot (Figure 2A). To investigate the transcriptional differences between AML patients and healthy donors further, differential gene expression analysis was performed for each T‐cell subpopulation in AML compared to corresponding subpopulations in healthy controls. This identified 244/236, 107/112, and 55/37 genes expressed at higher/lower levels by CTLs, Tregs, and Th cells, respectively, in AML as compared to control (Figure 2B; Tables S2‐S4).

### 3.2 *TIM3* is expressed at higher levels by AML‐CTLs than control‐CTLs

**FIGURE 3:**
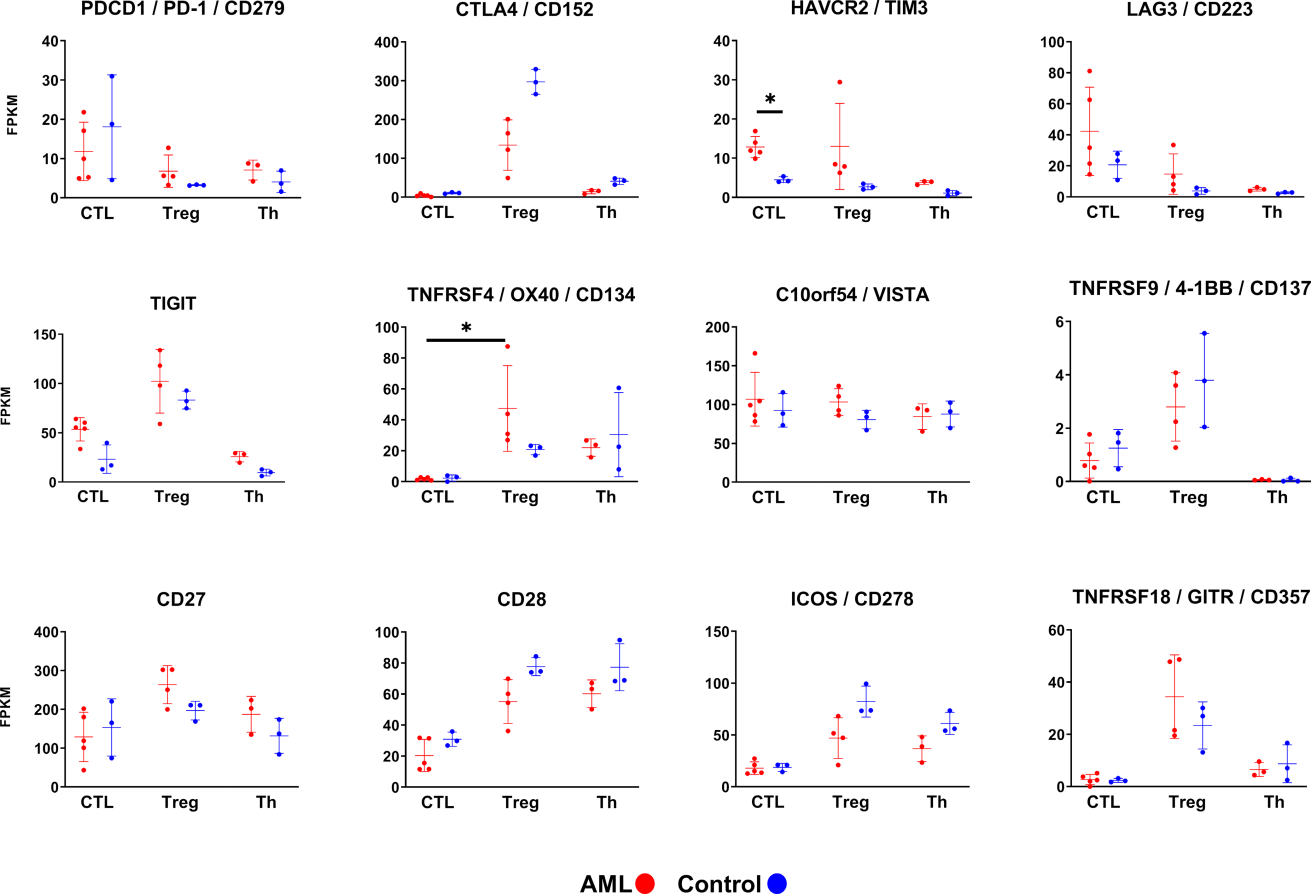
Expression levels for genes encoding established and emerging immuno‐oncological targets, by distinct T‐cell subpopulations in *TP53*‐mutated AML patients and healthy controls. Differences as compared to populations in AML were evaluated and considered significant at FDR < 0.05 (two tailed *t*‐test, based on all genes in the data set). Error bars represent mean ± SD; *FDR < 0.05. AML, acute myeloid leukemia; CTL, cytotoxic T‐cells; FDR, false discovery rate; Th, conventional helper T‐cells; Treg, regulatory T‐cells

To evaluate the potential of established and/or emerging immuno‐oncological drugs to affect T‐cells in *TP53*‐mutated AML, the expression levels of 12 molecules representing targets for such drugs were examined for the specific T‐cell subpopulations^20^, ^21^ (Figure 3). Notably, the exhaustion marker *TIM3* displayed a significantly higher expression in AML‐CTLs than control‐CTLs (FDR < 0.05), whereas no significant difference was detected in the expression of other molecules, including *PD‐1* and *CTLA‐4* that represent targets of approved drugs used for specific cancer patient groups. Also, *TNFRSF4/OX40* showed higher expression in AML‐Tregs as compared to AML‐CTLs (FDR < 0.05).

### 3.3 CTLs and Tregs display transcriptional signatures suggestive of altered metabolism in AML patients

GSEA was performed using the Hallmark gene sets to obtain a broad overview of pathways differentially regulated in specific T‐cell subpopulations in the AML cohort as compared to the respective population in healthy controls. CTLs, Tregs, and Th cells from AML patients were shown to be enriched for genes associated with fatty acid metabolism, as compared to their respective control subpopulations. (Table 2). AML‐Tregs were furthermore enriched for genes associated with glycolysis and oxidative phosphorylation as compared to control‐Tregs (Table 2), whereas these pathways were not shown to be enriched in AML‐CTLs as compared to control‐CTLs. Gene sets related to metabolism were furthermore shown to be enriched in AML‐Tregs versus AML‐CTLs, but not in the comparison of control‐Tregs to control‐CTLs (data not shown).

**TABLE 2:** GSEA results for different comparisons using the Hallmark gene sets

Comparison	Gene set title	Size	Matches	NES	FDR
AML‐CTLs vs Control‐CTLs	HALLMARK_INTERFERON_ALPHA_RESPONSE	97	92	2.01	**
	HALLMARK_INTERFERON_GAMMA_RESPONSE	200	196	1.79	**
	HALLMARK_FATTY_ACID_METABOLISM	158	156	1.78	**
	HALLMARK_XENOBIOTIC_METABOLISM	200	197	1.65	**
	HALLMARK_PROTEIN_SECRETION	96	95	1.53	*
	HALLMARK_TNFA_SIGNALING_VIA_NF‐κB	200	197	−1.79	*
	HALLMARK_HEDGEHOG_SIGNALING	36	35	−2.01	**
AML‐Tregs vs Control‐Tregs	HALLMARK_FATTY_ACID_METABOLISM	158	156	1.86	**
	HALLMARK_E2F_TARGETS	200	194	1.79	**
	HALLMARK_GLYCOLYSIS	200	197	1.66	**
	HALLMARK_OXIDATIVE_PHOSPHORYLATION	200	183	1.64	**
	HALLMARK_INTERFERON_ALPHA_RESPONSE	97	92	1.63	**
	HALLMARK_XENOBIOTIC_METABOLISM	200	197	1.61	*
	HALLMARK_MTORC1_SIGNALING	200	194	1.60	*
	HALLMARK_MYC_TARGETS_V2	58	58	1.54	*
	HALLMARK_INTERFERON_GAMMA_RESPONSE	200	196	1.49	*
	HALLMARK_G2M_CHECKPOINT	200	190	1.48	*
AML‐Th vs Control‐Th	HALLMARK_FATTY_ACID_METABOLISM	158	156	1.67	*
AML‐Tregs vs AML‐CTLs	HALLMARK_E2F_TARGETS	200	194	2.21	**
	HALLMARK_MYC_TARGETS_V1	200	193	2.19	**
	HALLMARK_G2M_CHECKPOINT	200	190	2.18	**
	HALLMARK_MITOTIC_SPINDLE	199	197	1.71	**
	HALLMARK_OXIDATIVE_PHOSPHORYLATION	200	183	1.69	**
	HALLMARK_FATTY_ACID_METABOLISM	158	156	1.55	*

*Note*: Statistically significant results from GSEA using the hallmark genes set are displayed. FDR <0.05 was considered significant (*FDR < 0.05 and **FDR < 0.01). Positive NES represents enrichment in the first as compared to the second subpopulation (column one).

Abbreviations: AML, acute myeloid leukemia; CTL, cytotoxic T‐cells; FDR, false discovery rate; GSEA, gene set enrichment analysis; NES, normalized enrichment score; Size, number of genes in the set; Th, conventional helper T‐cells.

In addition to metabolic pathways, AML‐Tregs were found to upregulate cell cycle pathways (E2F target and G2M checkpoint signatures) as compared to control‐Tregs as well as compared to AML‐CTLs. Moreover, AML‐CTLs and AML‐Tregs were both enriched for genes associated with IFN‐α and IFN‐γ responses, as compared to the corresponding population in healthy controls. Furthermore, AML‐CTLs displayed a negative enrichment score for gene sets associated with hedgehog signaling and NF‐κB mediated TNF‐signaling, as compared to the corresponding control subpopulation.

### 3.4 AML‐CTLs and AML‐Th cells show transcriptional features of exhaustion

**FIGURE 4:**
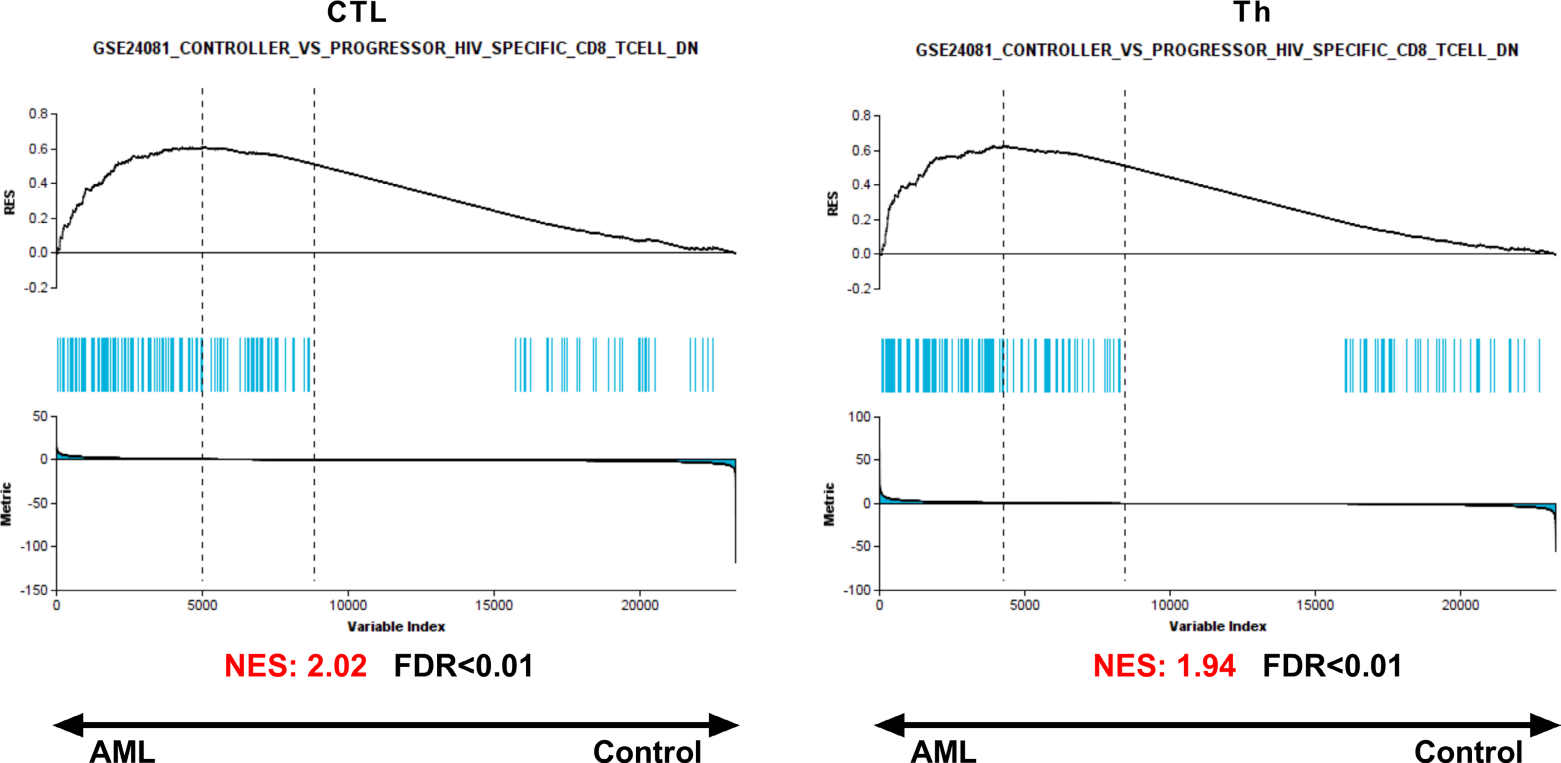
Results of GSEA performed to elucidate whether the applied gene set is statistically overrepresented/enriched among the down or upregulated genes when comparing AML‐CTLs and AML‐Th cells to their corresponding control from healthy individuals. The gene set comprises genes upregulated in human CTLs during chronic infection (GSE24081), as a model for T‐cell exhaustion. AML, acute myeloid leukemia; AML‐CTL, cytotoxic T‐cells from AML patients; AML‐Th, conventional helper T‐cells from AML patients; GSEA, gene set enrichment analysis; NES, normalized enrichment score

To investigate the exhaustion status of T‐cells in *TP53*‐mutated AML, we performed GSEA using three different exhaustion signatures, i.e., a signature of exhausted CD8^+^ T‐cells during chronic infection (CD8^+^ T‐cells in progressive HIV patients) and signatures of exhausted CD8^+^ T‐cells infiltrating liver and melanoma tumors, respectively.^17^, ^18^, ^19^ Compared to their respective subpopulations from healthy controls, AML‐CTLs and AML‐Th cells displayed a significant enrichment of genes associated with exhausted CD8^+^ T‐cells during chronic infection; however, significant enrichment was not observed for signatures based on exhausted CD8^+^ T‐cells from solid tumors (Table 3; Figure 4).

**TABLE 3:** Results from GSEA using three different exhaustion gene signatures

Comparison	Gene set title	Size	Matches	NES	FDR
AML‐CTLs vs Control‐CTLs	GSE24081_controller_vs_progressor_HIV_specific_CD8_Tcell_DN	196	183	2.02	**
	Exhausted CD8^+^ T‐cells in liver cancer patients‐Zheng et al.	82	78	1.07	NS
	Exhausted CD8^+^ T‐cells in Melanoma patients‐Tirosh et al.	132	132	1.00	NS
AML Th vs Control‐Th	GSE24081_controller_vs_progressor_HIV_specific_CD8_Tcell_DN	196	183	1.94	**
	Exhausted CD8^+^ T‐cells in liver cancer patients‐Zheng et al.	82	78	1.34	NS
	Exhausted CD8^+^ T‐cells in Melanoma patients‐Tirosh et al.	132	132	0.97	NS

*Note*: FDR < 0.05 was considered significant (**FDR < 0.01; NS, not significant). Positive NES represents enrichment in the first as compared to the second subpopulation (column one).

Abbreviations: AML, acute myeloid leukemia; CTL, cytotoxic T‐cells; FDR, false discovery rate; GSEA, gene set enrichment analysis; NES, normalized enrichment score; Size, number of genes in the set; Th, conventional helper T‐cells.

## 4 DISCUSSION

During the last decades, therapy based on blockade of PD‐1/PD‐L1 and/or CTLA‐4 has had a remarkable impact on survival in many cancers, but the effect in hematological malignancies such as AML has been limited.^5^ Interestingly, recent data showed that samples from AML patients with *TP53* mutation, a patient group with very poor prognosis,^9^, ^10^ display features associated with response to immunotherapy.^11^, ^12^ To gain further knowledge on T‐cells in *TP53*‐mutated AML, we performed detailed transcriptomic analyses of specific T‐cell subpopulations from this patient group and compared to corresponding populations from healthy controls. The results showed that in *TP53*‐mutated AML, CTLs and Th cells display gene expression signatures related to exhaustion as compared to control, while Tregs display signatures suggestive of altered metabolic programs, possibly enabling them to adapt to limited energy resources more effectively than CTLs.

We observed that AML‐CTLs, as well as AML‐Tregs from patients with *TP53* mutation, demonstrate signatures associated with IFN‐α and IFN‐γ signaling compared to the corresponding population from healthy controls. The increased interferon signaling indicates that the T‐cells are exposed to IFN‐α and IFN‐γ in the *TP53‐*mutated AML. Interestingly, an IFN‐γ‐dominant profile of AML patients' bone marrow samples was recently found to correlate with *TP53* mutation, and the results herein thus support and further extend these findings.^11^, ^12^

The presented data also show that AML‐CTLs display transcriptional alterations suggestive of exhaustion/dysfunction. For example, AML‐CTLs were significantly enriched for a gene set related to exhaustion during chronic infection in humans,^17^ as compared to healthy control, and this is in line with previous data based on CTLs in AMLs of different molecular subtypes.^4^ In contrast, CTLs in *TP53*‐mutated AML showed no enrichment of signatures related to exhausted CTLs in melanoma or in liver cancer, as compared to CTLs from healthy controls. This may suggest that different exhaustion programs are active in AML as compared to solid tumors. Additionally, we found a higher expression of *TIM3* in AML‐CTLs compared to control‐CTLs, further supporting dysfunctional or terminally exhausted CTLs.^22^, ^23^ Similarly, higher expression of TIM3 by AML‐CTLs in peripheral blood has previously been shown in a larger cohort of newly diagnosed patients, with various mutations, as compared to controls.^24^ TIM3 is a potential drug target in several cancers, as blockade can reactivate exhausted T‐cells, and trials in AML are currently ongoing.^25^ Of note, as *TIM3*, but not genes encoding other well‐known exhaustion markers such as *PD‐1*, *CTLA4*, *LAG3*, or *TIGIT*, was expressed at higher levels in *TP53*‐mutated AML than in controls, it could suggest that blockade of *TIM3* is of specific clinical relevance for boosting T‐cell activity in this patient group. AML‐CTLs from patients with various/not specified mutations have previously been shown to display features of exhaustion.^4^, ^26^, ^27^, ^28^ For example, a recent study using flow cytometry showed a higher frequency of PD‐1^+^ CTLs in AML bone marrow aspirates, and a higher frequency of these cells co‐expressed TIM3 or LAG3 as compared to their controls, thus supporting an exhausted profile.^28^ This is in line with the higher levels of *TIM3* observed for AML‐CTLs in the present study, but levels of *PD‐1* and *LAG3* were unaltered. In a study performing transcriptional profiling of different molecular subtypes of AML‐CTLs, upregulation of inhibitory molecules (*CD244*, *CD160*, *LILRB1*, *CD300A*, *LAG3*, *TIGIT*, *PVRIG*) and downregulation of stimulatory molecules (*CD40LG*, *CD28*, *ICOS*, *TNFSF8*, *TMIGD2*, *TNFRSF25*) were identified as compared to CTLs from healthy controls.^4^ In contrast, these were not differentially expressed in the present study focusing specifically on *TP53*‐mutated AML. One could thus speculate that the exhaustion profile of CTLs may be distinct in *TP53*‐mutated AML as compared to AML with other mutations.

The broad pathway analysis, using GSEA and the Hallmark gene sets, suggested that Tregs from patients with *TP53* mutation have different metabolic features in comparison to control‐Tregs and/or to AML‐CTLs. AML‐Tregs showed enrichment of gene sets associated with glycolysis, fatty acid metabolism, and oxidative phosphorylation as compared to controls, thus suggesting that AML‐Tregs upregulate their production of energy based on both glucose and fatty acids in the AML‐setting. Interestingly, both glycolysis and fatty acid metabolism were recently suggested to be important for the expansion of Tregs in cancer, based on studies in a murine colon carcinoma model.^29^ In line with this notion, GSEA of AML‐Tregs also showed enrichment of genes associated with cell cycle processes, such as E2F gene targets and G2/M checkpoint,^30^ thus supporting a proliferative status of AML‐Tregs compared to control‐Tregs. Of note, a higher frequency of AML‐Tregs was not observed as compared to control in our cohort (Figure S1). Nonetheless, this may be due to the limited number of donors as higher Treg frequency has previously been demonstrated in the bone marrow and blood of AML patients.^28^, ^31^ Furthermore, by comparing AML‐Tregs to AML‐CTLs, pathways related to oxidative phosphorylation, fatty acid metabolism, and cell cycle were shown to be enriched in AML‐Tregs. In conclusion, these observations suggest a superior metabolic adaptation by AML‐Tregs, as compared to AML‐CTLs, which can give them a functional and proliferative advantage over other T‐cells in *TP53‐*mutated AML, possibly supporting the poor disease outcome.

Relevant limitations to consider in the current study include the limited size of the cohort and the patient group comprising de novo, secondary and treatment‐related AML. Still, clear transcriptomic differences were observed between *TP53*‐mutated AML and healthy controls while applying stringent FDR (<0.05). Furthermore, to assess whether the observed differences are specific to *TP53*‐mutated AML or a characteristic feature also of other molecular subtypes of AML, additional studies investigating larger cohorts of AMLs are required. In addition, functional assessment of the T cell compartment in TP53‐mutated AML is needed to firmly establish altered functionality.

In summary, we demonstrate altered transcriptional profiles of CTLs, Tregs, and Th cells in *TP53‐*mutated AML, as compared to healthy controls, providing clues on mechanisms involved in the compromised immune response in this subtype of AML. The results suggest that AML‐Tregs alter their metabolic pathways and gain a metabolic advantage over AML‐CTLs. At the same time, AML‐CTLs appear to be functionally impaired, featuring exhaustion properties including higher expression of *TIM3*. Further exploration of these findings could hence open up for new therapeutic strategies for the treatment of *TP53*‐mutated AML, a subtype associated with dismal prognosis using current treatment regimens.

## CONFLICT OF INTEREST

Thoas Fioretos is a scientific advisor and board member of Qlucore AB (Ideon, Lund, Sweden).

The rest of the authors declare no financial or commercial conflict of interest.

## AUTHOR CONTRIBUTION

*Involved in cell sorting, analyzed the data, produced figures, and drafted the manuscript*: Milad Abolhalaj. *Involved in data analysis and manuscript revision*: Viktor Sincic. *Planned RNA extraction and sequencing and performed bioinformatical processing of the data*: Henrik Lilljebjörn. *Involved in antibody panel development and cell sorting*: Carl Sandén. *Supported data analysis, contributed with bioinformatical consultation*: Alar Aab. *Involved in antibody panel development and cell sorting*: Karin Hägerbrand. *Contributed to the study design*: Peter Ellmark. *Contributed to the study design*: Carl A. K. Borrebaeck. *Contributed to the study design and with patient samples*: Thoas Fioretos. *Contributed to the study design, involved in antibody panel development and cell sorting, supervised data analysis as well as manuscript drafting*: Kristina Lundberg. All authors contributed to the interpretation of results and were involved in analysis consultation as well as in the reviewing and editing of the manuscript. All authors approved the final version of the manuscript.

## ETHICAL APPROVAL STATEMENT

The research was performed in accordance with relevant guidelines/regulations and was approved by the Regional Ethics Committee (EPN‐Regionala Etikprövningsnämnden i Lund) and informed consent was obtained from all AML patients.

## Supporting information


FigureS 1

TableS 1

TableS 2

TableS 3

TableS 4

## Data Availability

Data available on request from the authors.
