# Supplementary material for: Transcriptional profiling demonstrates altered characteristics of CD8 + cytotoxic T‐cells and regulatory T‐cells in TP53‐mutated acute myeloid leukemia
Source: Cancer Med. 2022 Mar 16;11(15):3023–32. doi: 10.1002/cam4.4661 (PMC9359873; doi:10.1002/cam4.4661)
Supplement: Supplementary file 1 — FigureS 1 [file CAM4-11-3023-s005.docx]

**Supporting figure 1**


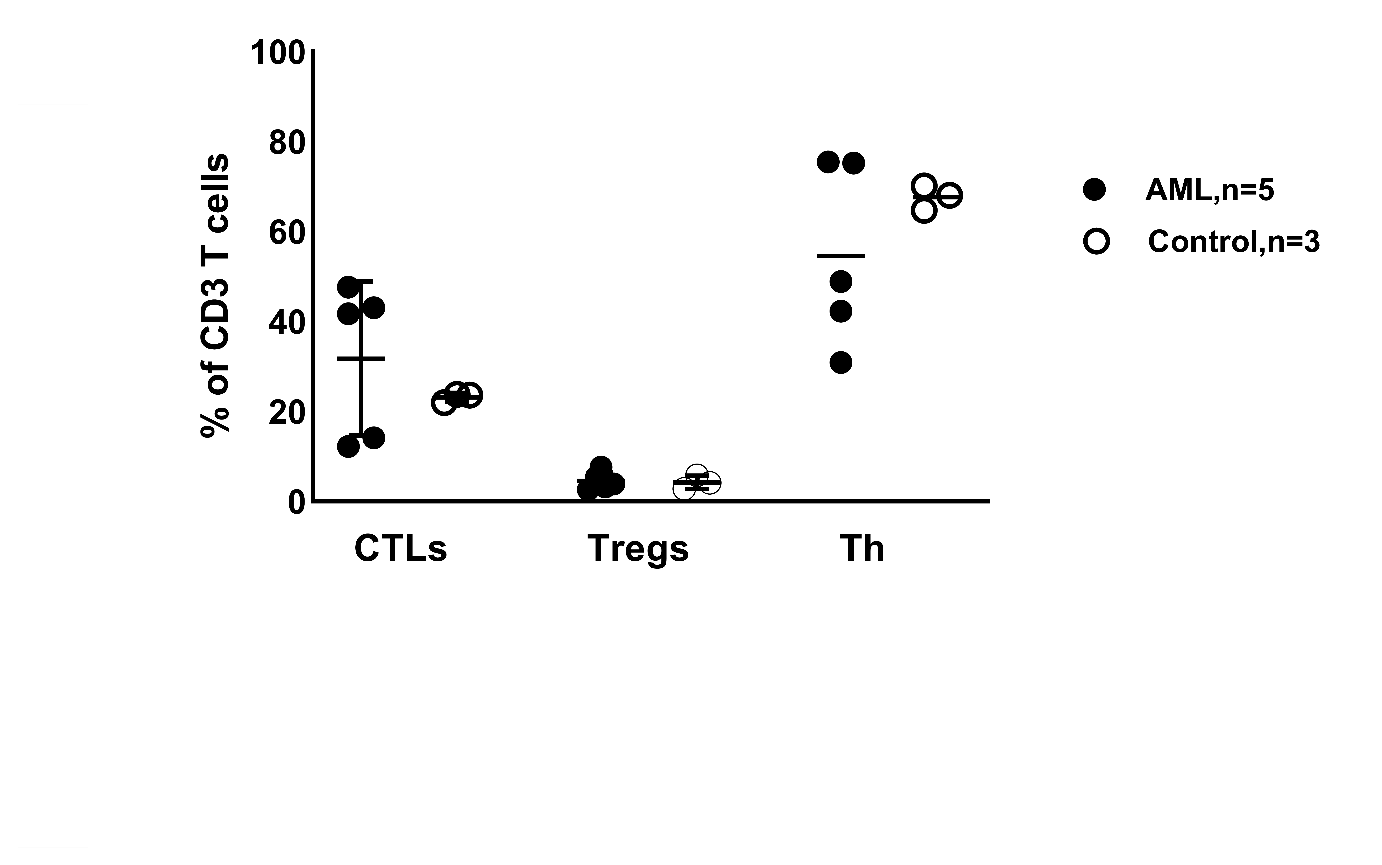


Frequency of CTLs, Tregs and Th cells in AML patients with TP53 mutation and in control subjects.

Abbreviations: AML = Acute myeloid leukemia; CTL= cytotoxic T-cells; Th = conventional helper T-cells; Treg =

regulatory T-cells.
