# Supplementary material for: Transcriptional profiling demonstrates altered characteristics of CD8 + cytotoxic T‐cells and regulatory T‐cells in TP53‐mutated acute myeloid leukemia
Source: Cancer Med. 2022 Mar 16;11(15):3023–32. doi: 10.1002/cam4.4661 (PMC9359873; doi:10.1002/cam4.4661)
Supplement: Supplementary file 2 — TableS 1 [file CAM4-11-3023-s002.docx]

**Supporting table 1**

Clinical information of the patients.

| **Gender** | **Disease** | **FAB** | **ELN** | **Karyotype** | **Mutations** |
| --- | --- | --- | --- | --- | --- |
| M | AML diagnosis | M5 | HR | Complex1 | TP53, FLT3 |
| F | tAML diagnosis | M2 | tAML | Complex2 | TP53 |
| M | sAML diagnosis | M2 | sAML | Complex3 | TP53, U2AF1 |
| F | AML diagnosis | M4 | HR | Complex4 | TP53, DNMT3A, |
| M | sAML diagnosis | M2 | sAML | Complex5 | TP53, TET2, NF1 |

Abbreviations; sAML: secondary AML, tAML: therapy-related AML, ELN: European Leukemia net risk classification 2017, LR: low risk, IR: intermediate risk, HR: high risk

**Complex1:** 47,XY,+6,+i(8)(q10),-18,-22,+mar/45-46,XY,-3,+add(6)(p21),+i(8)(q10),der(16)t(3;16)(p12;q11),-22

**Complex2**:43-47,XX,del(5)(q13q33),der(8)t(8;12)(p22;p13),add(11)(p15),-12,add(13)(p11),-18,del(20)(q11),add(22)(q13),+mar

**Complex3:**44,XY,-5,del(7)(q21),add(12)(p13),-17,add(17)(p13),ins(21;?)(q22;?)

**Complex4:**46,XX,der(3)t(1;3)(p13;q27),5,add(7)(q22),der(16)t(?5;16)(q?;p11),der(17)t(5;17)(q?;q21),der(21)t(11;21)(q13;q22),+mar

**Complex5:**45,XY,der(13;14)(q10;q10)/42-44,idem,-5,-7,-9,-10,-11,?hsr(11)(q23),-14,-16,-20,+4mar
