# Supplementary material for: Transcriptional profiling demonstrates altered characteristics of CD8 + cytotoxic T‐cells and regulatory T‐cells in TP53‐mutated acute myeloid leukemia
Source: Cancer Med. 2022 Mar 16;11(15):3023–32. doi: 10.1002/cam4.4661 (PMC9359873; doi:10.1002/cam4.4661)
Supplement: Supplementary file 3 — TableS 2 [file CAM4-11-3023-s003.docx]

**Supporting table 2**

Differentially expressed genes by AML CTL vs. control CTL.

| **Gene name** | **FDR<0.05** | **Fold change** | **Expression levels (log2 FPKM values)** | | | | | | | |
| --- | --- | --- | --- | --- | --- | --- | --- | --- | --- | --- |
|  |  |  | **AML-A** | **AML-B** | **AML-C** | **AML-D** | **AML-E** | **Normal-A** | **Normal-B** | **Normal-C** |
| OSCAR | 0.018088 | 420.0170172 | -0.21978 | 4.1759 | 1.6653 | 2.7263 | 2.0043 | -6.6439 | -6.6439 | -6.6439 |
| EPDR1 | 0.00408 | 340.2615978 | 2.4128 | 2.324 | 1.5518 | 0.3617 | 2.1827 | -6.6439 | -6.6439 | -6.6439 |
| HOMER3 | 0.020893 | 260.8360512 | -0.019907 | 3.8347 | 0.96402 | 2.0653 | 0.071378 | -6.6439 | -6.6439 | -6.6439 |
| CMTM2 | 0.018068 | 192.2226639 | 0.12159 | 3.3329 | 0.21389 | 0.38706 | 0.65823 | -6.6439 | -6.6439 | -6.6439 |
| RASGRP4 | 0.034628 | 184.3729931 | -1.2877 | 3.7287 | 0.40744 | 1.1748 | 0.38968 | -6.6439 | -6.6439 | -6.6439 |
| CD300LB | 0.002774 | 179.0382691 | 1.4004 | 1.5458 | -0.16128 | 0.79231 | 0.62389 | -6.6439 | -6.6439 | -6.6439 |
| LRP3 | 0.018816 | 113.3058516 | -1.4816 | 1.7285 | -0.71351 | 0.2704 | 1.0971 | -6.6439 | -6.6439 | -6.6439 |
| ESM1 | 0.041632 | 86.0703232 | -2.7891 | -1.0004 | 1.3017 | 0.67902 | 0.7265 | -6.6439 | -6.6439 | -6.6439 |
| SLC22A16 | 0.032169 | 83.43612823 | -0.82689 | 1.5441 | 0.84865 | -0.56056 | -2.3118 | -6.6439 | -6.6439 | -6.6439 |
| STBD1 | 0.043004 | 79.49726429 | 1.7147 | 0.6094 | 1.7224 | 0.39621 | -0.13771 | -6.6439 | -6.6439 | -3.0677 |
| TREML2 | 0.037057 | 73.81243353 | 0.63123 | 1.5832 | -0.1532 | -0.23967 | 1.3239 | -6.6439 | -6.6439 | -3.4423 |
| C11orf45 | 0.01088 | 72.82128819 | -0.57451 | -1.2704 | -1.0694 | -0.30793 | 0.93418 | -6.6439 | -6.6439 | -6.6439 |
| IL5RA | 0.019538 | 69.84296219 | 0.98304 | 0.71565 | -0.40342 | 0.44998 | -0.03387 | -6.6439 | -6.6439 | -4.0635 |
| LOC100507392 | 0.030612 | 60.73479425 | -2.1181 | 0.40915 | -1.0268 | -1.8521 | 0.99061 | -6.6439 | -6.6439 | -6.6439 |
| DNASE1L2 | 0.011404 | 59.18263914 | -0.56993 | -1.2147 | -0.47871 | -1.8889 | 0.36825 | -6.6439 | -6.6439 | -6.6439 |
| MYOT | 0.012333 | 50.58547219 | -1.6585 | -1.741 | 0.017195 | -1.3922 | -0.14174 | -6.6439 | -6.6439 | -6.6439 |
| C9orf139 | 0.018088 | 43.13344709 | -0.043864 | -1.8738 | -0.58056 | -2.5464 | -1.0212 | -6.6439 | -6.6439 | -6.6439 |
| LARGE | 0.014405 | 34.22304375 | -1.9754 | -0.28232 | -1.3004 | -1.7088 | -2.4681 | -6.6439 | -6.6439 | -6.6439 |
| CISH | 0.002774 | 28.74554228 | 6.9657 | 7.2514 | 6.7074 | 7.6779 | 7.3011 | 2.6432 | 2.3682 | 1.9949 |
| FZD2 | 0.006508 | 21.98610192 | -2.8528 | -1.9654 | -1.7628 | -2.5862 | -1.7597 | -6.6439 | -6.6439 | -6.6439 |
| PRH2 | 0.028173 | 21.19253404 | -2.0417 | -2.1571 | -2.9517 | -3.3033 | -0.73828 | -6.6439 | -6.6439 | -6.6439 |
| OAS1 | 0.043004 | 15.09556966 | 7.4308 | 4.9261 | 5.2742 | 5.5524 | 6.3946 | 1.6699 | 2.4185 | 1.9103 |
| S100A11 | 0.018088 | 10.65117011 | 9.5004 | 9.7503 | 8.4717 | 8.3721 | 8.9542 | 5.5125 | 5.8634 | 5.4145 |
| CXCR7 | 0.012333 | 10.03009709 | 2.8636 | 2.5145 | 3.127 | 3.2313 | 3.6189 | 0.19209 | -0.43075 | -0.52695 |
| ZNF28 | 0.042724 | 8.564744028 | 3.8397 | 2.1144 | 2.7543 | 3.0875 | 3.4154 | -0.16877 | -0.68555 | 0.68587 |
| GIMAP8 | 0.018068 | 8.221794245 | 4.9749 | 4.8492 | 5.2598 | 5.7765 | 4.9417 | 2.6393 | 1.5392 | 2.1844 |
| ZNF613 | 0.040834 | 7.857580202 | 2.2676 | 1.7205 | 2.2463 | 3.4749 | 2.7082 | 0.095735 | -0.52779 | -1.0397 |
| LPAR6 | 0.01404 | 7.328308917 | 5.459 | 5.5439 | 5.7645 | 5.5401 | 5.7139 | 2.1037 | 3.3537 | 2.735 |
| LOC729678 | 0.028928 | 6.788187787 | 4.009 | 4.2063 | 4.5779 | 3.9144 | 4.0314 | 2.1194 | 1.5138 | 0.52112 |
| TTC9C | 0.018068 | 6.682833447 | 4.359 | 3.5266 | 4.5064 | 4.1582 | 4.6386 | 1.7956 | 1.1836 | 1.5127 |
| ZNF616 | 0.043892 | 6.582887014 | 1.7429 | 1.2956 | 2.1701 | 2.5944 | 2.3972 | -1.3919 | -0.69632 | 0.052179 |
| C14orf119 | 0.007643 | 6.268411536 | 5.1663 | 4.8606 | 5.2601 | 5.4106 | 5.4289 | 2.7482 | 2.7626 | 2.2208 |
| GIMAP4 | 0.033762 | 6.24542545 | 8.9204 | 7.6847 | 8.666 | 9.0394 | 9.2155 | 6.3098 | 5.7533 | 6.1241 |
| ZNF175 | 0.04566 | 5.900389072 | 1.1151 | 1.0485 | 1.4603 | 1.2348 | 1.3695 | -1.1397 | -2.3525 | -0.45331 |
| TNFSF10 | 0.018088 | 5.734946074 | 5.4378 | 5.6544 | 5.042 | 5.4681 | 5.2551 | 3.4783 | 2.4413 | 2.6355 |
| C9orf64 | 0.049817 | 5.618467519 | 2.5497 | 2.6299 | 2.667 | 2.5801 | 3.0618 | 1.1031 | 0.30865 | -0.78918 |
| APOL1 | 0.030137 | 5.570448367 | 4.9549 | 3.6717 | 4.1216 | 4.2772 | 4.7539 | 1.6115 | 2.2882 | 1.7345 |
| C6orf211 | 0.002288 | 5.516933959 | 3.8892 | 4.1987 | 3.9055 | 4.0924 | 3.8862 | 1.7785 | 1.4166 | 1.3965 |
| ZNF781 | 0.041681 | 5.442705656 | 0.7693 | 0.92508 | 1.1225 | 0.80137 | 0.12697 | -0.85434 | -2.0659 | -2.1656 |
| THAP8 | 0.026738 | 5.442603119 | 2.4391 | 2.7497 | 2.4449 | 2.898 | 2.5267 | 0.41794 | -0.61816 | 0.70237 |
| C1GALT1C1 | 0.026284 | 5.345925991 | 4.5712 | 4.7873 | 4.7575 | 4.554 | 4.8437 | 2.5905 | 1.4677 | 2.7947 |
| SH2D3C | 0.032169 | 5.326175638 | 5.2956 | 4.5917 | 4.504 | 4.9216 | 5.8181 | 2.8892 | 2.3944 | 2.5557 |
| DCLRE1A | 0.015597 | 5.320481154 | 2.7663 | 2.5201 | 2.6847 | 2.5325 | 2.5853 | -0.39143 | 0.71686 | 0.29324 |
| STYK1 | 0.036972 | 5.216698257 | 1.4286 | 1.5859 | 2.2558 | 1.6462 | 2.5864 | -0.36529 | -0.9915 | -0.090881 |
| CETN3 | 0.032169 | 5.104843462 | 4.3359 | 4.2643 | 4.5181 | 5.0567 | 4.307 | 2.2845 | 1.4544 | 2.6947 |
| RAB10 | 0.009856 | 5.050874895 | 5.9307 | 6.4386 | 5.8016 | 6.0162 | 6.0044 | 3.6152 | 3.4677 | 4.0224 |
| HSCB | 0.003103 | 5.034796608 | 3.6524 | 3.614 | 3.2389 | 3.3167 | 3.629 | 1.2914 | 1.0025 | 1.1809 |
| ZNF232 | 0.04914 | 4.947878729 | 2.7717 | 2.2563 | 3.3796 | 3.1729 | 3.286 | 0.20892 | 0.39355 | 1.397 |
| METTL13 | 0.011404 | 4.79507469 | 3.9698 | 3.4031 | 4.0064 | 4.0667 | 4.2731 | 1.6061 | 1.716 | 1.7247 |
| MGC57346 | 0.042724 | 4.486042911 | 2.294 | 2.2412 | 2.2626 | 2.6729 | 3.1782 | -0.22068 | 0.37889 | 0.9348 |
| TMEM60 | 0.043386 | 4.476713716 | 5.1401 | 5.0221 | 5.1028 | 5.3583 | 5.6784 | 3.4998 | 2.2589 | 3.535 |
| NDUFAF1 | 0.005034 | 4.446345564 | 3.9576 | 4.4501 | 4.4228 | 4.2948 | 4.1468 | 1.9361 | 2.1741 | 2.1952 |
| ERLIN1 | 0.04078 | 4.405829285 | 3.2918 | 4.0686 | 4.2797 | 3.5094 | 4.1009 | 1.2354 | 2.2302 | 1.6664 |
| PRDX3 | 0.037883 | 4.390606242 | 6.3145 | 7.3836 | 6.4728 | 6.4537 | 6.452 | 4.665 | 3.964 | 4.8137 |
| PPIL1 | 0.026284 | 4.305836204 | 4.1333 | 5.0117 | 4.6823 | 4.258 | 4.138 | 1.9587 | 2.4639 | 2.5925 |
| GIMAP7 | 0.049087 | 4.302573429 | 9.3173 | 8.8465 | 9.7189 | 9.7659 | 9.7104 | 8.0527 | 6.8438 | 7.2033 |
| STX3 | 0.031676 | 4.209095286 | 2.4695 | 2.6032 | 2.4957 | 2.0767 | 2.6108 | 0.71524 | -0.32904 | 0.74681 |
| TRIB3 | 0.02887 | 4.17405614 | 3.139 | 3.1963 | 3.6671 | 3.5446 | 3.9913 | 0.94663 | 1.6441 | 1.7479 |
| PIGV | 0.018088 | 4.11103028 | 3.6214 | 3.3539 | 3.9588 | 3.6271 | 3.8053 | 1.7851 | 1.9535 | 1.1628 |
| CENPK | 0.044682 | 4.052802704 | 3.806 | 4.3606 | 4.4299 | 3.8723 | 3.8208 | 2.5915 | 1.3432 | 2.1823 |
| TMEM79 | 0.032102 | 4.009771461 | 2.9197 | 2.7021 | 2.7798 | 2.9558 | 2.9286 | 1.5588 | 0.28321 | 0.71903 |
| UEVLD | 0.018088 | 4.001174173 | 3.1096 | 2.795 | 2.8107 | 2.6501 | 2.8298 | 0.5961 | 1.3634 | 0.55635 |
| COPB2 | 0.018088 | 3.994440252 | 5.2799 | 5.5881 | 5.2825 | 5.1634 | 5.5249 | 3.7987 | 3.4284 | 2.8822 |
| MSL3P1 | 0.04914 | 3.966848992 | 1.339 | 1.0805 | 1.6519 | 1.4354 | 2.2076 | -0.72176 | -0.759 | 0.14542 |
| TRNT1 | 0.04566 | 3.942106391 | 4.0731 | 3.7898 | 4.2734 | 3.8056 | 4.5226 | 2.0648 | 2.763 | 1.514 |
| ZNF691 | 0.018088 | 3.92409545 | 3.2159 | 3.298 | 3.6513 | 3.4547 | 3.6539 | 1.622 | 1.0028 | 1.8224 |
| SAC3D1 | 0.01088 | 3.612433917 | 2.0854 | 2.2956 | 2.0451 | 2.0759 | 2.1685 | 0.25271 | 0.63396 | -0.043283 |
| ZNF696 | 0.032169 | 3.568439745 | 1.6182 | 1.0417 | 2.0142 | 1.7473 | 1.7012 | -0.04766 | 0.02388 | -0.60854 |
| BET1 | 0.026809 | 3.545363265 | 3.4067 | 3.1628 | 3.9268 | 3.5226 | 3.6271 | 2.0604 | 1.782 | 1.2674 |
| TNFRSF1A | 0.032169 | 3.477008695 | 4.3377 | 5.3624 | 4.5056 | 4.771 | 5.0337 | 3.1438 | 3.0304 | 2.8385 |
| CRIPT | 0.018816 | 3.475659778 | 3.183 | 3.5345 | 3.7288 | 3.7805 | 3.4993 | 1.3375 | 1.9637 | 1.9426 |
| STT3A | 0.031676 | 3.460163068 | 4.9469 | 5.5102 | 4.7705 | 4.718 | 5.4756 | 3.0229 | 3.4868 | 3.3705 |
| TMEM99 | 0.026809 | 3.441768251 | 2.6329 | 2.78 | 3.1541 | 2.4631 | 2.2088 | 1.032 | 0.60216 | 0.95973 |
| SLC25A44 | 0.026029 | 3.437786794 | 3.1189 | 3.3606 | 3.8139 | 3.5674 | 3.7986 | 1.7306 | 2.1031 | 1.4175 |
| MINPP1 | 0.045075 | 3.431644315 | 2.519 | 2.7387 | 2.6073 | 2.99 | 3.1952 | 0.42842 | 1.5338 | 1.1312 |
| PGBD2 | 0.048974 | 3.40729488 | 3.8975 | 3.4705 | 3.9521 | 4.1446 | 3.4571 | 2.4059 | 2.2526 | 1.3887 |
| DCAF12 | 0.020156 | 3.391868391 | 3.4074 | 3.696 | 3.8752 | 3.823 | 4.1918 | 2.3664 | 1.8643 | 1.8791 |
| CEPT1 | 0.045075 | 3.328241328 | 3.929 | 4.3638 | 4.5234 | 4.6421 | 4.8195 | 2.7525 | 2.2401 | 3.1698 |
| TRMT1L | 0.015006 | 3.302274295 | 2.9463 | 2.6847 | 3.1379 | 3.4141 | 3.0863 | 1.4382 | 1.3809 | 1.1721 |
| MED11 | 0.023942 | 3.266713577 | 5.158 | 4.8322 | 5.2341 | 5.2118 | 4.6656 | 3.2475 | 2.994 | 3.696 |
| CDK2AP2 | 0.026951 | 3.261825887 | 6.9186 | 6.8191 | 6.6516 | 6.6672 | 7.2409 | 4.7157 | 5.2206 | 5.5251 |
| STIP1 | 0.021402 | 3.257142242 | 5.0731 | 5.4052 | 5.1473 | 5.1437 | 5.2279 | 2.9753 | 3.7227 | 3.7895 |
| ACTR3 | 0.018088 | 3.241421565 | 7.9701 | 8.363 | 7.8383 | 7.6401 | 8.0938 | 6.194 | 6.1147 | 6.5446 |
| PLSCR1 | 0.027979 | 3.237260439 | 5.5817 | 5.1354 | 4.5828 | 4.8137 | 5.0686 | 3.2401 | 3.3943 | 3.3906 |
| PSMA5 | 0.018816 | 3.138002873 | 5.1427 | 5.3593 | 4.8015 | 4.7272 | 5.3047 | 3.4877 | 3.2086 | 3.5554 |
| CCDC99 | 0.043892 | 3.134003513 | 2.3176 | 2.0606 | 2.3477 | 2.584 | 1.7377 | 0.13687 | 0.99704 | 0.55063 |
| RPE | 0.043418 | 3.113146553 | 3.6045 | 4.2292 | 3.9249 | 3.384 | 3.7526 | 2.0819 | 1.749 | 2.5911 |
| TMEM165 | 0.018088 | 3.101085707 | 5.5689 | 5.972 | 5.4699 | 5.2824 | 5.6705 | 4.1812 | 3.7426 | 3.9561 |
| AARS | 0.019602 | 3.087516237 | 4.6775 | 4.6083 | 3.8924 | 4.3858 | 4.5184 | 2.7502 | 2.8822 | 2.7377 |
| EIF2B2 | 0.046655 | 3.051996885 | 4.3843 | 4.8213 | 4.6581 | 4.4559 | 5.1095 | 3.1549 | 3.5033 | 2.57 |
| DUSP18 | 0.049621 | 3.022397367 | 3.6675 | 4.1625 | 3.8198 | 3.6089 | 3.2776 | 2.5898 | 1.7138 | 2.0311 |
| CLCN3 | 0.037413 | 3.019619294 | 2.5637 | 2.5546 | 3.1379 | 2.9844 | 2.5719 | 1.1155 | 0.7903 | 1.5986 |
| ILK | 0.033623 | 3.006780936 | 6.5961 | 6.6067 | 6.2323 | 5.9152 | 6.3463 | 4.3502 | 4.8676 | 5.0355 |
| HCG11 | 0.04941 | 2.994911666 | 1.0062 | 1.6358 | 1.3915 | 1.5948 | 1.9834 | -0.16699 | 0.35531 | -0.36884 |
| TRIM46 | 0.021633 | 2.988245371 | 0.77366 | 0.613 | 1.0123 | 1.2902 | 1.0606 | -0.3571 | -0.66132 | -0.86962 |
| ALDH1B1 | 0.015597 | 2.982061557 | 2.6928 | 2.9003 | 2.931 | 2.3646 | 2.8758 | 1.2969 | 0.97007 | 1.2628 |
| ARV1 | 0.015597 | 2.973935812 | 4.2982 | 4.4639 | 4.8768 | 4.4401 | 4.2417 | 3.0508 | 2.8294 | 2.7951 |
| CDC42EP3 | 0.013987 | 2.970021374 | 5.87 | 5.8374 | 6.005 | 5.8442 | 6.2191 | 4.5822 | 4.4952 | 4.0766 |
| DCAF7 | 0.013915 | 2.96923935 | 4.5206 | 4.3805 | 4.7744 | 4.3663 | 4.745 | 3.1245 | 2.7354 | 3.1019 |
| CCDC56 | 0.032448 | 2.965687869 | 6.2135 | 6.146 | 6.2884 | 5.7416 | 6.345 | 4.6773 | 4.1348 | 4.9235 |
| G3BP1 | 0.007643 | 2.927833305 | 6.2024 | 5.8679 | 6.1569 | 6.0751 | 6.2467 | 4.4341 | 4.7421 | 4.5037 |
| LMAN2L | 0.012604 | 2.926737346 | 3.877 | 3.5633 | 3.8322 | 3.6533 | 3.89 | 2.4349 | 2.2853 | 1.9214 |
| MCM3 | 0.026809 | 2.875275342 | 5.4712 | 5.918 | 5.0527 | 5.3946 | 5.565 | 4.0023 | 3.7697 | 4.0978 |
| SLC36A1 | 0.034628 | 2.86950147 | 0.32954 | 1.1564 | 0.89742 | 0.91816 | 0.83533 | -1.0395 | -0.5318 | -0.50899 |
| LEO1 | 0.018024 | 2.861146198 | 4.4806 | 4.2934 | 4.4584 | 4.4722 | 4.9862 | 2.9634 | 2.9924 | 3.1089 |
| NUPL2 | 0.02039 | 2.848850727 | 4.6571 | 4.1831 | 4.6926 | 4.2453 | 4.0613 | 2.765 | 2.9921 | 2.8154 |
| MOB3A | 0.009856 | 2.848363727 | 6.1821 | 5.8293 | 5.9025 | 5.9834 | 6.2017 | 4.425 | 4.4061 | 4.6979 |
| HAVCR2 | 0.028173 | 2.835677259 | 3.5774 | 3.3061 | 3.5248 | 4.0817 | 3.8048 | 2.0483 | 2.4239 | 1.9936 |
| PCGF6 | 0.037749 | 2.821404173 | 3.4973 | 3.4559 | 2.8017 | 2.9253 | 3.6492 | 1.745 | 1.7949 | 1.7685 |
| ZMYM6NB | 0.02787 | 2.816987694 | 7.416 | 6.968 | 7.5318 | 7.2203 | 7.1405 | 6.1083 | 5.4054 | 5.7698 |
| VPS35 | 0.025566 | 2.815283195 | 5.3472 | 5.8091 | 5.8949 | 5.4 | 5.7112 | 4.458 | 4.065 | 3.8946 |
| GEMIN5 | 0.037883 | 2.794286694 | 2.4919 | 2.2021 | 1.9012 | 2.6693 | 2.3174 | 0.54518 | 1.1958 | 0.76072 |
| KIAA0040 | 0.018088 | 2.791976434 | 5.0966 | 4.6596 | 4.7009 | 4.9371 | 5.1034 | 3.5612 | 3.1414 | 3.5521 |
| GART | 0.027122 | 2.751503375 | 5.103 | 5.0009 | 4.8856 | 4.9146 | 5.227 | 3.9312 | 3.1212 | 3.6456 |
| C12orf4 | 0.022473 | 2.747577247 | 2.5222 | 3.079 | 3.0446 | 2.7176 | 3.0129 | 1.654 | 1.1574 | 1.4399 |
| ALAS1 | 0.035828 | 2.738868541 | 4.3636 | 4.2913 | 4.2696 | 4.2194 | 4.6465 | 2.3882 | 3.169 | 3.1563 |
| CCT5 | 0.045497 | 2.724604057 | 5.9222 | 6.6903 | 5.7407 | 5.8598 | 5.7989 | 4.6377 | 4.5591 | 4.4722 |
| SNAPIN | 0.037397 | 2.708936577 | 5.8225 | 6.2627 | 6.2821 | 6.1638 | 6.2572 | 4.2873 | 4.7253 | 5.1472 |
| C14orf142 | 0.044087 | 2.705071635 | 4.4973 | 4.8648 | 4.8041 | 4.2975 | 3.9733 | 3.2422 | 2.9342 | 2.9788 |
| RAB39B | 0.026809 | 2.698778638 | 2.6978 | 2.5705 | 3.3092 | 2.9812 | 3.101 | 1.6503 | 1.419 | 1.4296 |
| RNF34 | 0.02787 | 2.681202257 | 5.9582 | 5.4727 | 5.9837 | 5.8302 | 6.2281 | 4.6964 | 4.4728 | 4.2459 |
| MKKS | 0.012604 | 2.677228001 | 4.7081 | 4.8025 | 4.8793 | 4.9385 | 4.5713 | 3.1429 | 3.3363 | 3.5984 |
| CCDC47 | 0.049087 | 2.666190567 | 4.7263 | 4.8219 | 4.6549 | 4.41 | 4.6203 | 3.2122 | 3.7578 | 2.7257 |
| CCDC111 | 0.02979 | 2.658784265 | 3.6661 | 3.4151 | 3.3868 | 3.7187 | 3.8603 | 1.9607 | 2.0274 | 2.6078 |
| CPSF2 | 0.037413 | 2.639491193 | 3.7054 | 3.8928 | 3.7135 | 3.8351 | 4.2565 | 2.92 | 2.2908 | 2.2304 |
| CEP72 | 0.013987 | 2.631964625 | 1.718 | 1.8497 | 2.1598 | 1.8269 | 1.9573 | 0.29313 | 0.51678 | 0.70869 |
| ERI1 | 0.044087 | 2.63194005 | 2.9253 | 3.6251 | 3.1971 | 2.6337 | 3.1991 | 1.6186 | 1.8377 | 1.7035 |
| UMPS | 0.007643 | 2.629253852 | 2.6662 | 2.922 | 2.7325 | 2.8938 | 2.8241 | 1.6241 | 1.2636 | 1.3515 |
| UBXN2B | 0.032448 | 2.616660339 | 2.9563 | 3.3542 | 3.2256 | 3.0398 | 3.6919 | 1.6411 | 1.8769 | 2.0795 |
| SMARCAL1 | 0.014405 | 2.615922722 | 3.737 | 3.7638 | 3.9418 | 3.903 | 3.5865 | 2.1135 | 2.5194 | 2.5644 |
| TCHP | 0.032606 | 2.596245295 | 4.0171 | 3.6672 | 3.995 | 3.8028 | 3.9687 | 2.4414 | 2.957 | 2.1428 |
| GTF2H1 | 0.010323 | 2.589439791 | 4.0991 | 4.4051 | 4.3221 | 4.5592 | 4.4037 | 3.1173 | 2.8769 | 2.9614 |
| ARPC5 | 0.027561 | 2.582461564 | 8.1449 | 8.5363 | 7.9804 | 7.9645 | 8.3648 | 6.8083 | 6.5933 | 7.0867 |
| AHSA1 | 0.020073 | 2.581470355 | 5.9857 | 6.3022 | 6.0231 | 5.9781 | 6.2962 | 4.4106 | 4.8541 | 4.9819 |
| PIM1 | 0.043022 | 2.562609131 | 7.4332 | 7.8684 | 7.0939 | 7.4908 | 7.8681 | 6.2744 | 5.9465 | 6.3589 |
| FAM118B | 0.018088 | 2.548438095 | 3.4993 | 3.8815 | 3.3703 | 3.6601 | 3.3322 | 2.3696 | 2.1619 | 2.0657 |
| ALG10 | 0.041988 | 2.54765024 | 0.039756 | 0.51761 | 0.3523 | 0.30629 | 0.42393 | -1.4319 | -1.0585 | -0.57317 |
| GVINP1 | 0.036372 | 2.547260824 | 3.5506 | 3.549 | 3.7003 | 3.7734 | 3.8746 | 2.8187 | 2.2134 | 1.9898 |
| PRKAR1A | 0.024337 | 2.540466166 | 6.7602 | 6.8023 | 6.7238 | 6.6824 | 6.9821 | 5.1995 | 5.8667 | 5.269 |
| PSEN1 | 0.020073 | 2.515781949 | 3.6582 | 4.2526 | 4.1178 | 4.0281 | 4.1685 | 2.6266 | 2.9192 | 2.5963 |
| LASP1 | 0.040979 | 2.493075829 | 5.0327 | 4.6894 | 5.0651 | 5.0301 | 5.459 | 3.4172 | 3.9816 | 3.8132 |
| ARMC7 | 0.031268 | 2.480595856 | 2.4201 | 1.7779 | 2.0599 | 1.9139 | 1.9689 | 0.97982 | 0.7194 | 0.45314 |
| MLEC | 0.042724 | 2.472916042 | 3.5942 | 4.1526 | 3.7458 | 3.8898 | 3.269 | 2.3792 | 2.3094 | 2.5836 |
| MID1IP1 | 0.03621 | 2.460025706 | 4.1769 | 4.3149 | 4.6729 | 4.7171 | 4.0449 | 3.2565 | 3.0854 | 2.9181 |
| ATG4C | 0.03803 | 2.429669446 | 3.0791 | 3.1694 | 3.2732 | 3.3587 | 2.7634 | 1.9796 | 1.4785 | 2.0859 |
| CCDC75 | 0.043247 | 2.429635718 | 3.2596 | 2.9705 | 3.3509 | 3.291 | 3.1627 | 2.2094 | 1.425 | 2.1442 |
| HINFP | 0.041988 | 2.429399836 | 3.9362 | 3.9083 | 4.0496 | 4.139 | 3.9334 | 2.8949 | 2.1962 | 3.047 |
| LOC401397 | 0.012333 | 2.407129234 | 5.5289 | 5.6658 | 5.8996 | 5.5208 | 5.4963 | 4.4609 | 4.2235 | 4.3805 |
| PUS3 | 0.025476 | 2.406138916 | 3.4426 | 3.6009 | 3.8265 | 3.203 | 3.3941 | 2.308 | 2.3649 | 2.0072 |
| RNF20 | 0.015008 | 2.393751333 | 4.4571 | 4.2053 | 4.2448 | 4.1143 | 3.9112 | 2.8581 | 3.0292 | 2.8945 |
| SOCS2 | 0.033762 | 2.385492061 | 4.6939 | 4.5213 | 4.6378 | 4.221 | 4.7136 | 3.6654 | 3.0723 | 3.172 |
| WDR3 | 0.034922 | 2.366125588 | 2.9055 | 3.4374 | 3.4996 | 3.6854 | 3.4004 | 1.9936 | 2.2255 | 2.2103 |
| SNX1 | 0.008104 | 2.351780157 | 3.5569 | 3.7123 | 3.6684 | 3.5567 | 3.9088 | 2.5392 | 2.3777 | 2.4237 |
| DYNLL1 | 0.022325 | 2.34699236 | 7.9741 | 7.9788 | 7.6897 | 7.5455 | 8.0063 | 6.4436 | 6.5459 | 6.8347 |
| MAPK14 | 0.026951 | 2.334672567 | 5.0267 | 5.4689 | 5.3831 | 5.1481 | 5.0698 | 4.2716 | 3.9659 | 3.7508 |
| PTPN6 | 0.043004 | 2.331858313 | 7.3449 | 7.7146 | 7.5396 | 7.379 | 7.1738 | 6.0852 | 5.9313 | 6.6102 |
| RPA2 | 0.041239 | 2.320431931 | 7.4364 | 7.2755 | 7.1254 | 7.0635 | 7.8315 | 6.1365 | 6.0287 | 6.231 |
| C9orf23 | 0.015006 | 2.319885164 | 4.9928 | 5.0279 | 4.9488 | 5.022 | 5.1161 | 3.5466 | 3.79 | 4.0858 |
| PROSC | 0.02887 | 2.317527794 | 5.2616 | 5.5379 | 5.8882 | 5.6502 | 5.3287 | 4.3329 | 4.4677 | 4.1616 |
| C21orf49 | 0.049817 | 2.267318021 | 1.9228 | 2.3066 | 2.5148 | 2.3788 | 2.7211 | 1.4386 | 1.1058 | 1.0191 |
| VTA1 | 0.018088 | 2.22427213 | 4.7467 | 4.9692 | 4.8456 | 5.0866 | 5.0469 | 3.5339 | 3.9615 | 3.8616 |
| AHCYL1 | 0.015597 | 2.221416754 | 4.5724 | 4.4995 | 4.3792 | 4.2432 | 4.3101 | 3.044 | 3.2584 | 3.4458 |
| UTP6 | 0.012639 | 2.21177834 | 5.0102 | 5.2667 | 5.0328 | 5.0339 | 4.9636 | 4.0518 | 3.7048 | 3.9921 |
| FANCG | 0.037568 | 2.199699864 | 3.0461 | 2.623 | 2.5694 | 2.5453 | 2.6619 | 1.2956 | 1.8549 | 1.505 |
| KIAA0930 | 0.049097 | 2.172596188 | 2.7516 | 2.6119 | 2.3345 | 2.8078 | 3.1038 | 1.4826 | 1.8291 | 1.4958 |
| CASP4 | 0.047003 | 2.16122112 | 6.2889 | 6.5949 | 6.0329 | 5.9016 | 6.3831 | 4.9571 | 5.3303 | 5.0979 |
| ESRRA | 0.027397 | 2.15615359 | 3.5161 | 3.1464 | 3.5748 | 3.2813 | 3.4832 | 2.5624 | 2.1233 | 2.19 |
| M6PR | 0.026738 | 2.154122252 | 6.5852 | 6.6304 | 6.6512 | 6.5988 | 6.9909 | 5.4515 | 5.4374 | 5.8637 |
| HSPA8 | 0.008736 | 2.148505504 | 9.6739 | 9.8231 | 9.7059 | 9.5419 | 9.8607 | 8.5425 | 8.5919 | 8.7189 |
| NOC3L | 0.049817 | 2.138609933 | 3.0097 | 3.0068 | 2.8088 | 3.1608 | 2.9881 | 2.067 | 1.4399 | 2.1876 |
| WDR11 | 0.034922 | 2.130935155 | 3.8922 | 3.5861 | 4.2461 | 3.9012 | 3.787 | 2.6643 | 2.9798 | 2.729 |
| MGAT1 | 0.04778 | 2.12751125 | 6.1936 | 6.3454 | 6.0947 | 6.2273 | 6.5875 | 4.8151 | 5.4067 | 5.3798 |
| CCDC117 | 0.018816 | 2.126773916 | 3.7965 | 3.6268 | 3.9731 | 3.5776 | 4.033 | 2.7819 | 2.7186 | 2.6377 |
| CCND2 | 0.041239 | 2.100927597 | 5.9416 | 5.6203 | 6.3015 | 5.6993 | 5.9031 | 4.8229 | 4.9401 | 4.7034 |
| ASH2L | 0.015597 | 2.099850693 | 4.5746 | 4.3673 | 4.2246 | 4.3174 | 4.5647 | 3.339 | 3.4734 | 3.2059 |
| UBFD1 | 0.025186 | 2.095827563 | 2.9089 | 2.7497 | 3.2154 | 3.0919 | 3.2992 | 2.0042 | 2.0272 | 1.9251 |
| AP3M1 | 0.012604 | 2.090816788 | 4.1077 | 4.0511 | 4.3176 | 3.9379 | 4.0982 | 3.0908 | 3.1278 | 2.8967 |
| HNRNPF | 0.018088 | 2.071419965 | 7.5665 | 7.4335 | 7.4182 | 7.3382 | 7.7792 | 6.3259 | 6.5706 | 6.473 |
| SLC35A5 | 0.033623 | 2.060707776 | 4.1355 | 3.6153 | 3.7803 | 3.997 | 4.2106 | 2.9488 | 2.92 | 2.845 |
| SLC39A1 | 0.041988 | 2.048602324 | 4.5929 | 4.5737 | 4.0293 | 4.1291 | 4.4827 | 3.2481 | 3.4258 | 3.3068 |
| MTERFD1 | 0.04941 | 2.043374172 | 4.459 | 5.0548 | 4.8803 | 4.9416 | 4.8664 | 3.5493 | 4.065 | 3.8141 |
| UTP11L | 0.034628 | 2.029784466 | 4.1323 | 4.4403 | 4.0318 | 3.8469 | 4.265 | 3.038 | 3.0343 | 3.2935 |
| ECI2 | 0.042724 | 2.027431877 | 5.788 | 5.7284 | 5.5717 | 6.0362 | 6.0298 | 5.0688 | 4.5576 | 4.8071 |
| SELPLG | 0.038195 | 2.020828231 | 8.4278 | 7.8813 | 8.2669 | 8.323 | 8.5534 | 7.3523 | 7.236 | 7.2383 |
| CNPY4 | 0.046307 | 2.015428639 | 3.802 | 3.5111 | 4.156 | 4.1087 | 3.8658 | 2.865 | 2.7449 | 3.023 |
| RSPRY1 | 0.015597 | 1.999667526 | 4.0949 | 4.0817 | 4.208 | 4.4706 | 4.3211 | 3.3033 | 3.1781 | 3.2251 |
| GPKOW | 0.032448 | 1.9630997 | 3.933 | 3.7304 | 3.7204 | 3.3685 | 3.6717 | 2.7744 | 2.8263 | 2.5343 |
| FXR2 | 0.028928 | 1.961494476 | 3.2263 | 3.0052 | 3.059 | 3.3622 | 2.8434 | 2.0983 | 2.0127 | 2.2708 |
| RAC2 | 0.026951 | 1.951234106 | 9.2078 | 9.2112 | 9.1825 | 8.8662 | 9.3144 | 7.9907 | 8.2463 | 8.3391 |
| TMX2 | 0.041632 | 1.939601374 | 5.7185 | 5.9258 | 6.1621 | 5.6354 | 5.8885 | 4.7249 | 4.8808 | 5.1252 |
| MCFD2 | 0.033959 | 1.938633434 | 3.6874 | 4.0922 | 4.1416 | 3.8207 | 3.9583 | 3.1995 | 2.8458 | 2.9097 |
| NCOA4 | 0.018375 | 1.937773653 | 6.6495 | 6.9587 | 6.5568 | 6.7104 | 6.8141 | 5.6885 | 5.9307 | 5.7313 |
| TMEM14A | 0.045075 | 1.934025491 | 4.8754 | 4.3792 | 4.839 | 4.695 | 4.9186 | 3.5872 | 3.8085 | 3.9738 |
| AASDHPPT | 0.020073 | 1.928742047 | 4.8551 | 4.7926 | 5.1303 | 5.069 | 5.2068 | 4.115 | 3.9746 | 4.0997 |
| PEF1 | 0.045075 | 1.923943941 | 5.9365 | 5.9993 | 6.1057 | 5.6466 | 6.2779 | 4.8981 | 5.2024 | 5.0469 |
| C21orf59 | 0.041254 | 1.915383496 | 5.8794 | 5.7618 | 5.7235 | 5.6721 | 5.6332 | 4.5788 | 4.6498 | 5.1605 |
| DPAGT1 | 0.026284 | 1.911078168 | 4.5583 | 4.9007 | 4.6328 | 4.8087 | 4.4471 | 3.7151 | 3.8594 | 3.6309 |
| TXNDC9 | 0.034922 | 1.907963219 | 5.6626 | 5.5188 | 5.441 | 5.6945 | 5.2916 | 4.8159 | 4.5809 | 4.3722 |
| ING4 | 0.043892 | 1.90404371 | 5.6648 | 5.6761 | 5.8586 | 5.6157 | 5.8573 | 4.8448 | 4.4738 | 5.0977 |
| BRD8 | 0.043108 | 1.899684679 | 4.7527 | 4.3255 | 4.5032 | 4.8047 | 4.8667 | 3.7373 | 3.5861 | 3.851 |
| IL27RA | 0.041579 | 1.898333254 | 5.3072 | 5.2313 | 5.3823 | 5.3303 | 5.7679 | 4.4822 | 4.3127 | 4.6423 |
| NCAPD3 | 0.044339 | 1.898307138 | 2.6326 | 3.1605 | 3.0572 | 2.9312 | 2.9304 | 2.2373 | 2.0073 | 1.8084 |
| MRS2 | 0.019538 | 1.894775356 | 3.8961 | 3.5977 | 3.6873 | 3.9821 | 3.8126 | 2.7484 | 3.0059 | 2.8651 |
| STX6 | 0.019373 | 1.89409231 | 3.4403 | 3.4972 | 3.7438 | 3.5618 | 3.5526 | 2.7825 | 2.4168 | 2.7136 |
| TMBIM4 | 0.04914 | 1.886144415 | 6.1975 | 6.177 | 6.2635 | 6.2316 | 6.4231 | 5.0109 | 5.3363 | 5.6821 |
| MOCS2 | 0.018816 | 1.871817766 | 4.9313 | 4.9592 | 5.121 | 4.7044 | 4.7923 | 3.8971 | 4.0552 | 4.0393 |
| EIF2S1 | 0.034628 | 1.866065952 | 4.8093 | 5.0702 | 4.7122 | 4.9785 | 4.8888 | 4.1743 | 4.079 | 3.7221 |
| FNTA | 0.012333 | 1.862490811 | 6.4537 | 6.5421 | 6.628 | 6.6911 | 6.5526 | 5.7939 | 5.7157 | 5.5192 |
| C21orf33 | 0.021855 | 1.859928115 | 5.6634 | 5.7297 | 6.0495 | 5.8492 | 5.8411 | 4.7555 | 5.0228 | 5.0157 |
| PARP1 | 0.04914 | 1.849668376 | 5.6018 | 5.8604 | 5.7152 | 5.5411 | 5.7625 | 4.9043 | 4.4698 | 5.0527 |
| NAT10 | 0.048121 | 1.845681333 | 4.0496 | 3.9914 | 4.0993 | 4.2876 | 4.2482 | 3.5333 | 2.9406 | 3.2793 |
| ZNF568 | 0.045595 | 1.840860156 | 2.1469 | 2.4894 | 2.7093 | 2.622 | 2.6958 | 1.5519 | 1.6986 | 1.7064 |
| ACTR1A | 0.047403 | 1.840077567 | 5.5436 | 5.1825 | 5.0517 | 5.275 | 5.6087 | 4.3996 | 4.5409 | 4.4171 |
| GOT2 | 0.043386 | 1.819270249 | 5.7956 | 5.6885 | 5.5618 | 5.5228 | 5.8506 | 4.7312 | 4.62 | 5.1103 |
| USO1 | 0.033623 | 1.819253563 | 4.376 | 4.7232 | 4.7081 | 4.507 | 4.3716 | 3.6145 | 3.5479 | 3.8591 |
| GLT8D1 | 0.035756 | 1.803542495 | 5.5558 | 5.4613 | 5.3537 | 5.4101 | 5.7821 | 4.8305 | 4.4779 | 4.6769 |
| NBR1 | 0.033959 | 1.794000212 | 5.3695 | 5.0264 | 5.1089 | 5.2797 | 5.2199 | 4.5678 | 4.3666 | 4.1387 |
| ZNF641 | 0.042724 | 1.777241791 | 3.3336 | 3.3344 | 3.6321 | 3.2332 | 3.0934 | 2.3429 | 2.5501 | 2.5941 |
| BFAR | 0.042441 | 1.775665806 | 4.1835 | 4.1852 | 3.9795 | 3.7748 | 4.1968 | 3.365 | 3.2876 | 3.0542 |
| PDHA1 | 0.009856 | 1.754175656 | 4.5507 | 4.7875 | 4.5283 | 4.6462 | 4.6611 | 3.785 | 3.8022 | 3.8847 |
| GOSR2 | 0.035779 | 1.75249059 | 4.7763 | 4.4743 | 4.7351 | 4.7468 | 5.0222 | 3.9415 | 4.0045 | 3.8786 |
| HERC4 | 0.037057 | 1.73305027 | 4.0767 | 3.8798 | 4.1293 | 3.8175 | 3.8581 | 3.205 | 3.329 | 2.9429 |
| UNC45A | 0.02787 | 1.719814789 | 4.2355 | 4.1132 | 4.2552 | 4.1614 | 4.2983 | 3.3031 | 3.2995 | 3.6888 |
| DSCR3 | 0.010188 | 1.715338551 | 5.4363 | 5.3844 | 5.3856 | 5.5919 | 5.4701 | 4.698 | 4.7517 | 4.5758 |
| MESDC2 | 0.048332 | 1.714538132 | 4.5528 | 4.7846 | 4.9032 | 4.5753 | 4.4367 | 3.76 | 4.0432 | 3.8149 |
| KDELC2 | 0.022325 | 1.701357921 | 3.1123 | 3.2047 | 3.0637 | 2.9016 | 3.1703 | 2.2476 | 2.4936 | 2.2303 |
| ICMT | 0.026809 | 1.700233984 | 3.2214 | 3.4849 | 3.3113 | 3.4796 | 3.4208 | 2.5832 | 2.8053 | 2.4651 |
| TCEAL8 | 0.04768 | 1.696592464 | 5.5268 | 5.7629 | 5.6751 | 5.7859 | 5.6265 | 4.8249 | 4.7086 | 5.2049 |
| FAM48A | 0.008104 | 1.678570871 | 5.0794 | 5.1837 | 4.9488 | 5.0178 | 5.0668 | 4.3219 | 4.2541 | 4.3602 |
| TOLLIP | 0.038224 | 1.667515285 | 3.8918 | 3.8287 | 3.8873 | 4.0335 | 3.6632 | 3.2335 | 2.9084 | 3.2277 |
| C4orf34 | 0.03537 | 1.665636253 | 4.3258 | 4.6757 | 4.4894 | 4.3634 | 4.3954 | 3.8965 | 3.6554 | 3.5897 |
| DCUN1D1 | 0.024337 | 1.663128857 | 3.7823 | 3.8587 | 4.1692 | 3.9758 | 3.871 | 3.1408 | 3.196 | 3.2557 |
| C5orf22 | 0.018816 | 1.640300334 | 3.5634 | 3.7507 | 3.8064 | 3.6889 | 3.9009 | 3.0747 | 2.948 | 3.0616 |
| HIRIP3 | 0.018088 | 1.636930885 | 3.4624 | 3.6641 | 3.6069 | 3.7139 | 3.625 | 3.0523 | 2.8544 | 2.8037 |
| ABHD3 | 0.042724 | 1.592286454 | 5.0759 | 5.1795 | 5.2063 | 5.3988 | 5.4575 | 4.6291 | 4.4674 | 4.681 |
| XRCC5 | 0.020454 | 1.567226033 | 6.8434 | 7.0539 | 6.8283 | 6.7977 | 6.9281 | 6.3625 | 6.1496 | 6.2141 |
| DNAJC5 | 0.035828 | 1.549174936 | 2.9744 | 3.041 | 2.9123 | 3.0587 | 2.8591 | 2.3535 | 2.5141 | 2.1452 |
| NVL | 0.040979 | 1.547536409 | 3.715 | 3.5287 | 3.8827 | 3.8541 | 3.6387 | 3.0852 | 3.195 | 3.0014 |
| SARS | 0.026551 | 1.530440202 | 6.7999 | 6.6982 | 6.6769 | 6.7261 | 6.9108 | 6.1938 | 6.2517 | 5.9998 |
| DDX23 | 0.028928 | 1.517812165 | 5.5519 | 5.6922 | 5.6793 | 5.3747 | 5.6612 | 5.0398 | 4.9612 | 4.9686 |
| RDH11 | 0.043399 | 1.496556246 | 5.3731 | 5.2179 | 5.3009 | 5.4309 | 5.4651 | 4.758 | 4.6167 | 4.9531 |
| FGFR1OP2 | 0.014287 | 1.474691746 | 5.8938 | 5.9134 | 6.0365 | 5.9217 | 6.0325 | 5.4686 | 5.3086 | 5.4203 |
| NSUN2 | 0.03249 | 1.448473531 | 5.1754 | 4.9869 | 5.146 | 5.2676 | 5.1466 | 4.7056 | 4.6326 | 4.4917 |
| PDHB | 0.045497 | 1.434405906 | 6.041 | 5.9847 | 5.9506 | 6.023 | 6.1193 | 5.3868 | 5.7101 | 5.4129 |
| SRP14 | 0.032031 | 0.745944429 | 8.5377 | 8.5504 | 8.4117 | 8.5176 | 8.6223 | 8.8547 | 8.9634 | 9.0343 |
| NPLOC4 | 0.018279 | 0.727527376 | 5.0282 | 4.8885 | 4.8514 | 4.8121 | 4.88 | 5.3387 | 5.3196 | 5.3946 |
| RMND5A | 0.038049 | 0.727090454 | 4.3561 | 4.427 | 4.4371 | 4.431 | 4.313 | 4.706 | 4.9962 | 4.8557 |
| PPIG | 0.037057 | 0.722902696 | 5.227 | 5.1401 | 5.1966 | 5.326 | 5.381 | 5.8147 | 5.7177 | 5.6344 |
| MAP4K5 | 0.042724 | 0.713220432 | 3.4994 | 3.2092 | 3.4404 | 3.3471 | 3.394 | 3.8083 | 3.9769 | 3.8116 |
| RIOK1 | 0.042724 | 0.713200661 | 4.0147 | 4.2733 | 4.119 | 4.1837 | 4.3037 | 4.6051 | 4.643 | 4.7514 |
| LSM14A | 0.011884 | 0.711416624 | 6.0755 | 6.0814 | 6.0486 | 6.1024 | 6.1686 | 6.6321 | 6.6323 | 6.4952 |
| KTN1 | 0.044339 | 0.708798142 | 4.6204 | 4.4643 | 4.6636 | 4.6017 | 4.5644 | 5.2563 | 5.0253 | 4.9567 |
| POLR2D | 0.018088 | 0.695396056 | 4.4194 | 4.4817 | 4.5874 | 4.5079 | 4.6223 | 5.1285 | 4.9915 | 5.0235 |
| CYTIP | 0.04914 | 0.691055405 | 6.7369 | 6.9455 | 6.887 | 7.131 | 7.0218 | 7.5117 | 7.4416 | 7.4794 |
| SRSF11 | 0.045075 | 0.686923004 | 6.4761 | 6.3965 | 6.6447 | 6.5244 | 6.7449 | 7.1631 | 7.1143 | 7.0199 |
| ARGLU1 | 0.034628 | 0.685153688 | 7.6769 | 7.575 | 7.6912 | 7.8824 | 7.6795 | 8.1435 | 8.2496 | 8.3464 |
| CHD2 | 0.049817 | 0.683582134 | 5.9018 | 5.7383 | 5.9702 | 5.991 | 5.7873 | 6.6029 | 6.3308 | 6.3459 |
| THUMPD1 | 0.047089 | 0.678151747 | 5.4109 | 5.3999 | 5.2815 | 5.4393 | 5.1588 | 6.0253 | 5.9274 | 5.7425 |
| TNKS2 | 0.042662 | 0.677659935 | 4.3372 | 4.4506 | 4.4123 | 4.3439 | 4.547 | 5.0061 | 5.1345 | 4.7981 |
| ZC3H7A | 0.034922 | 0.657103955 | 4.8137 | 4.8687 | 4.7343 | 4.958 | 4.8901 | 5.4917 | 5.6123 | 5.2723 |
| PCNP | 0.041988 | 0.654957528 | 6.6818 | 6.5822 | 6.974 | 6.6618 | 6.8239 | 7.3592 | 7.4091 | 7.2975 |
| CMPK1 | 0.021165 | 0.643661242 | 6.8273 | 6.5904 | 6.7362 | 6.6616 | 6.8442 | 7.3499 | 7.2809 | 7.4719 |
| ITM2B | 0.021868 | 0.638907984 | 8.7997 | 9.0924 | 8.991 | 8.9338 | 8.9975 | 9.6241 | 9.4945 | 9.709 |
| PSMD7 | 0.022028 | 0.634321792 | 6.3984 | 6.3203 | 6.3049 | 6.2059 | 6.3576 | 6.8574 | 7.1573 | 6.9077 |
| UBE2G2 | 0.041988 | 0.628312038 | 5.7221 | 6.0434 | 5.825 | 5.832 | 5.6656 | 6.6019 | 6.3297 | 6.5326 |
| C19orf6 | 0.037413 | 0.627717246 | 6.0431 | 5.9417 | 6.3219 | 6.1862 | 6.3167 | 6.8073 | 6.8485 | 6.8454 |
| SNRPB2 | 0.034628 | 0.623505183 | 5.3997 | 5.2575 | 5.322 | 5.0688 | 5.0907 | 5.975 | 5.9772 | 5.7756 |
| HNRPDL | 0.018088 | 0.621382447 | 6.6714 | 6.7961 | 6.6313 | 6.7119 | 6.5109 | 7.307 | 7.2896 | 7.4557 |
| DAZAP2 | 0.012333 | 0.61798046 | 8.9033 | 8.9177 | 8.8305 | 8.8395 | 8.931 | 9.4917 | 9.7285 | 9.5161 |
| RB1CC1 | 0.037397 | 0.616337842 | 4.0047 | 3.8363 | 4.0172 | 4.0795 | 3.9426 | 4.9043 | 4.6265 | 4.492 |
| DIS3 | 0.035828 | 0.60496909 | 3.1578 | 2.9946 | 3.321 | 3.1528 | 3.2453 | 4.0555 | 3.9474 | 3.6952 |
| ZC3HAV1 | 0.026551 | 0.603893723 | 6.1728 | 6.1943 | 6.1353 | 6.3835 | 6.1596 | 7.1332 | 6.868 | 6.809 |
| MATR3 | 0.023926 | 0.603380526 | 6.7931 | 6.7101 | 6.7247 | 6.8946 | 6.6597 | 7.5229 | 7.6375 | 7.2955 |
| WDR26 | 0.026809 | 0.598299566 | 3.1978 | 3.2262 | 3.1698 | 3.0282 | 2.9382 | 3.8612 | 3.9981 | 3.7 |
| PPIL4 | 0.012604 | 0.59678638 | 3.4272 | 3.6609 | 3.6151 | 3.5255 | 3.5619 | 4.3379 | 4.189 | 4.3816 |
| SF3B1 | 0.043892 | 0.595846712 | 7.3689 | 7.6815 | 7.6771 | 7.879 | 7.4864 | 8.4138 | 8.3779 | 8.305 |
| POLE3 | 0.024571 | 0.592297297 | 6.1658 | 6.3256 | 6 | 6.0856 | 5.9173 | 6.9047 | 6.8318 | 6.8269 |
| LLPH | 0.018068 | 0.591195261 | 5.7423 | 5.6153 | 5.6854 | 5.5361 | 5.8506 | 6.3432 | 6.4816 | 6.5079 |
| STIM2 | 0.023645 | 0.588891393 | 4.2648 | 3.9901 | 4.2534 | 4.2711 | 4.1968 | 5.1323 | 4.8942 | 4.851 |
| VAPA | 0.007832 | 0.588537645 | 4.7889 | 4.8711 | 4.7258 | 4.825 | 4.8804 | 5.4688 | 5.6222 | 5.6581 |
| ZNF44 | 0.045075 | 0.584796542 | 3.8774 | 3.7407 | 3.9658 | 4.015 | 3.7658 | 4.7933 | 4.7825 | 4.365 |
| TFAM | 0.017826 | 0.583239309 | 4.4328 | 4.4119 | 4.533 | 4.7279 | 4.4972 | 5.2294 | 5.2856 | 5.3802 |
| HNRNPH1 | 0.026951 | 0.580480718 | 7.3497 | 7.202 | 6.9395 | 7.3383 | 7.1206 | 7.9379 | 7.9583 | 8.0279 |
| MAGED1 | 0.031484 | 0.576489721 | 4.5977 | 4.5286 | 4.4331 | 4.3108 | 4.6778 | 5.1142 | 5.3702 | 5.4283 |
| TBC1D15 | 0.011301 | 0.572330297 | 3.378 | 3.3305 | 3.1507 | 3.4317 | 3.2832 | 4.142 | 4.0517 | 4.166 |
| CCNI | 0.021017 | 0.570872318 | 6.9504 | 7.3063 | 6.9746 | 7.0415 | 6.9049 | 7.8557 | 7.7948 | 7.8824 |
| ANAPC2 | 0.018105 | 0.570242203 | 2.4115 | 2.5356 | 2.2333 | 2.5939 | 2.3646 | 3.3193 | 3.2148 | 3.1803 |
| WBP11 | 0.018088 | 0.566698541 | 5.8434 | 5.7669 | 5.6747 | 5.9281 | 5.9345 | 6.6118 | 6.8131 | 6.5217 |
| PARP6 | 0.019046 | 0.565450668 | 4.3854 | 4.4716 | 4.5248 | 4.4449 | 4.5785 | 5.257 | 5.1329 | 5.5208 |
| TERF2IP | 0.026809 | 0.563890339 | 7.0885 | 6.8255 | 7.1682 | 6.9992 | 7.2082 | 7.7339 | 7.9137 | 8.0057 |
| RIOK2 | 0.048671 | 0.563059737 | 3.8463 | 4.1367 | 3.6725 | 3.8824 | 4.0354 | 4.9734 | 4.5721 | 4.6844 |
| DUS3L | 0.04941 | 0.560230866 | 4.3685 | 4.1115 | 4.0592 | 4.3955 | 3.9631 | 5.0664 | 5.1756 | 4.8044 |
| SF1 | 0.027979 | 0.556083494 | 5.7819 | 5.8965 | 6.0726 | 6.1218 | 5.8854 | 6.8107 | 6.9718 | 6.6123 |
| PTPMT1 | 0.049817 | 0.554092927 | 4.3504 | 4.7086 | 4.5961 | 4.4196 | 4.5263 | 5.1113 | 5.3448 | 5.6599 |
| HNRNPL | 0.029854 | 0.553732034 | 4.8677 | 4.5326 | 4.5704 | 4.5152 | 4.5044 | 5.2635 | 5.4822 | 5.6067 |
| RCL1 | 0.03904 | 0.553586233 | 3.5696 | 3.6379 | 3.3303 | 3.8046 | 3.6735 | 4.35 | 4.3502 | 4.6687 |
| SUMO3 | 0.020073 | 0.550024012 | 6.1526 | 6.1071 | 5.7918 | 6.064 | 6.16 | 6.8091 | 6.9164 | 7.0271 |
| FOXN3 | 0.049087 | 0.545241206 | 3.9125 | 3.8086 | 4.0388 | 4.1991 | 4.364 | 4.9689 | 5.0695 | 4.7805 |
| EIF5 | 0.027794 | 0.543922614 | 5.8817 | 6.1305 | 5.7502 | 6.1562 | 5.8301 | 6.8517 | 6.9132 | 6.7199 |
| KRT10 | 0.029854 | 0.53797833 | 5.15 | 5.0577 | 5.1425 | 5.2956 | 5.0578 | 6.2548 | 5.7533 | 6.0972 |
| C9orf78 | 0.021402 | 0.537590729 | 6.8377 | 6.9942 | 7.0452 | 6.8678 | 7.118 | 7.665 | 7.8893 | 8.0497 |
| PDCL3 | 0.049817 | 0.536796227 | 5.3542 | 5.2222 | 5.0877 | 5.0502 | 5.3291 | 5.7884 | 6.3888 | 6.1415 |
| DDHD2 | 0.011884 | 0.534813095 | 4.4373 | 4.3227 | 4.2754 | 4.3862 | 4.2776 | 5.2251 | 5.4095 | 5.0936 |
| MED21 | 0.022028 | 0.534395671 | 4.2585 | 3.8492 | 4.1248 | 4.227 | 4.2149 | 4.9912 | 5.1667 | 4.9588 |
| RBM7 | 0.024282 | 0.531482266 | 4.1722 | 4.5007 | 4.4801 | 4.6457 | 4.3891 | 5.3271 | 5.248 | 5.4733 |
| ZNF622 | 0.018816 | 0.530716547 | 5.6241 | 5.8391 | 5.508 | 5.809 | 5.8037 | 6.5013 | 6.6148 | 6.7762 |
| MRPS30 | 0.033375 | 0.527611083 | 5.5467 | 5.5656 | 5.2171 | 5.4548 | 5.4717 | 6.2612 | 6.2078 | 6.6519 |
| ZNF207 | 0.026809 | 0.525790512 | 6.5761 | 6.8244 | 6.357 | 6.3954 | 6.6499 | 7.3945 | 7.5889 | 7.4806 |
| NGDN | 0.043399 | 0.525185898 | 5.8481 | 5.8285 | 5.7958 | 6.0127 | 5.9614 | 6.5139 | 6.7783 | 7.163 |
| QRICH1 | 0.029039 | 0.524850988 | 5.1807 | 4.7453 | 5.0753 | 5.0428 | 4.9383 | 5.8842 | 6.1325 | 5.7628 |
| NUFIP2 | 0.021855 | 0.523547833 | 2.8155 | 2.7302 | 3.1554 | 2.8711 | 2.7581 | 3.8847 | 3.8498 | 3.6645 |
| ITGAE | 0.028928 | 0.52184021 | 2.1528 | 2.5094 | 2.4873 | 2.1784 | 2.098 | 3.1519 | 3.3586 | 3.16 |
| BCLAF1 | 0.028515 | 0.521587117 | 5.3889 | 5.4527 | 5.4445 | 5.5994 | 5.3684 | 6.4096 | 6.6507 | 6.1091 |
| FAM76B | 0.020073 | 0.52015249 | 3.5465 | 3.2673 | 3.6771 | 3.4912 | 3.6906 | 4.3699 | 4.5706 | 4.4921 |
| DDX47 | 0.018088 | 0.519787289 | 5.587 | 5.5374 | 5.4738 | 5.7692 | 5.8759 | 6.544 | 6.6242 | 6.6098 |
| PAIP1 | 0.045075 | 0.516025209 | 4.2245 | 4.5013 | 4.1833 | 4.4731 | 3.8982 | 5.2335 | 5.0813 | 5.3169 |
| G3BP2 | 0.021868 | 0.515357872 | 6.0846 | 5.7679 | 6.2348 | 6.0971 | 5.8965 | 6.9744 | 7.0651 | 6.8781 |
| UNK | 0.015597 | 0.507366456 | 3.8694 | 4.2226 | 3.9065 | 3.9063 | 3.9602 | 5.0652 | 4.9593 | 4.8312 |
| CIR1 | 0.026551 | 0.507317198 | 4.6149 | 4.523 | 4.5588 | 4.8812 | 4.6104 | 5.5341 | 5.8702 | 5.4458 |
| RIOK3 | 0.016954 | 0.504192546 | 4.7222 | 4.5721 | 4.5591 | 4.74 | 4.4665 | 5.5966 | 5.7794 | 5.4238 |
| SFPQ | 0.03621 | 0.498456635 | 5.9995 | 6.2239 | 5.9664 | 5.8036 | 5.6283 | 6.8664 | 7.1056 | 6.8144 |
| PLEKHF2 | 0.011301 | 0.496394835 | 4.6871 | 4.5088 | 4.7361 | 4.8373 | 4.5945 | 5.6559 | 5.6016 | 5.7921 |
| MRPL44 | 0.018088 | 0.496339834 | 4.5404 | 4.6033 | 4.4825 | 4.5893 | 4.2015 | 5.4993 | 5.6091 | 5.3736 |
| TRIAP1 | 0.037912 | 0.493579165 | 5.4884 | 5.5671 | 5.8164 | 5.4812 | 5.23 | 6.5553 | 6.3143 | 6.7362 |
| HECA | 0.04768 | 0.490509621 | 5.0897 | 4.566 | 4.8833 | 5.1754 | 4.6342 | 6.0513 | 5.812 | 5.8288 |
| TOPORS | 0.047511 | 0.490158427 | 4.2294 | 4.2573 | 4.35 | 4.5466 | 4.4873 | 5.5751 | 5.6351 | 4.9982 |
| RPF1 | 0.041988 | 0.489510952 | 4.8886 | 4.8875 | 4.7797 | 5.2805 | 4.6506 | 5.7333 | 6.1071 | 5.9435 |
| COPS2 | 0.012604 | 0.486291482 | 3.4504 | 3.4045 | 3.3118 | 3.5342 | 3.2714 | 4.2946 | 4.6222 | 4.3869 |
| WTAP | 0.04941 | 0.48601298 | 6.4901 | 6.9555 | 6.6588 | 6.7346 | 6.622 | 7.3459 | 7.9424 | 7.9111 |
| PNRC1 | 0.027794 | 0.485413775 | 8.1421 | 8.1697 | 8.0388 | 8.559 | 7.97 | 9.2751 | 9.1557 | 9.2251 |
| PITPNC1 | 0.046307 | 0.482948107 | 5.9552 | 6.1346 | 6.1341 | 6.3157 | 5.9931 | 6.7769 | 7.4814 | 7.2115 |
| FLJ43663 | 0.033623 | 0.478930001 | 4.9477 | 4.4298 | 4.6244 | 4.6797 | 4.3875 | 5.6017 | 5.8739 | 5.5522 |
| PTMA | 0.037883 | 0.478797249 | 9.4669 | 9.6357 | 9.1716 | 9.4664 | 9.1985 | 10.26 | 10.348 | 10.743 |
| GTF2B | 0.049817 | 0.474947522 | 4.9566 | 5.4483 | 4.964 | 5.4544 | 5.4504 | 6.2949 | 6.1479 | 6.5439 |
| FASTKD5 | 0.045075 | 0.474857542 | 4.1541 | 3.7731 | 4.2895 | 4.3096 | 4.1137 | 5.4727 | 5.2183 | 4.9163 |
| HIST1H1E | 0.042685 | 0.47255911 | 3.8082 | 3.9117 | 3.4246 | 3.6583 | 3.3522 | 4.9398 | 4.6888 | 4.5087 |
| SAMD8 | 0.042685 | 0.47073705 | 2.4584 | 2.4379 | 2.2902 | 2.4032 | 2.0231 | 3.7526 | 3.1318 | 3.3443 |
| MED10 | 0.045641 | 0.469672405 | 6.5949 | 6.5867 | 6.4483 | 6.4441 | 6.7338 | 7.2064 | 7.8788 | 7.8703 |
| MAPK1IP1L | 0.012604 | 0.467347167 | 4.8931 | 4.9473 | 4.8341 | 4.8159 | 4.9421 | 5.7308 | 6.1446 | 6.0764 |
| C5orf24 | 0.029039 | 0.463628117 | 2.6559 | 2.4022 | 2.7102 | 2.5651 | 2.5418 | 4.0485 | 3.522 | 3.4815 |
| MYLIP | 0.018088 | 0.460266113 | 5.565 | 5.8641 | 5.6146 | 5.8315 | 6.0615 | 6.9904 | 6.8899 | 6.8401 |
| SMCHD1 | 0.018088 | 0.460166062 | 4.8795 | 4.5688 | 4.8344 | 4.9499 | 4.6947 | 5.8796 | 6.1148 | 5.7213 |
| CEBPZ | 0.031793 | 0.459974769 | 4.8184 | 4.6846 | 4.7934 | 5.056 | 4.7199 | 6.0234 | 6.19 | 5.5911 |
| LOC100507246 | 0.02787 | 0.458892071 | 5.0887 | 5.0718 | 4.8663 | 5.1226 | 5.3099 | 5.8952 | 6.3758 | 6.3759 |
| KLHDC2 | 0.002774 | 0.458059426 | 5.9231 | 6.0253 | 5.7752 | 5.9016 | 5.978 | 7.0048 | 7.0308 | 7.1055 |
| PHF12 | 0.037883 | 0.456491598 | 3.0566 | 2.5971 | 2.9062 | 3.3235 | 3.1944 | 4.1839 | 4.1962 | 4.0606 |
| RNMTL1 | 0.014405 | 0.451392186 | 4.3088 | 4.271 | 4.2295 | 4.575 | 4.6163 | 5.6193 | 5.5051 | 5.5186 |
| ATP6V1G1 | 0.034922 | 0.450198542 | 6.9934 | 7.3559 | 7.4283 | 7.5333 | 7.1031 | 8.3276 | 8.2696 | 8.7053 |
| ZBTB25 | 0.033623 | 0.450088295 | 4.4338 | 4.9529 | 4.6974 | 4.9048 | 4.953 | 6.2041 | 5.7999 | 5.8163 |
| SLC25A36 | 0.043386 | 0.448825515 | 3.3521 | 4.0848 | 3.835 | 3.8857 | 4.0792 | 5.1044 | 4.8816 | 5.0234 |
| KLHL24 | 0.03249 | 0.448597455 | 3.0055 | 3.0196 | 3.375 | 3.5415 | 3.3222 | 4.6453 | 4.2749 | 4.3076 |
| FBXO32 | 0.04566 | 0.446727421 | 3.4753 | 3.6366 | 3.8375 | 3.8295 | 3.0676 | 4.7048 | 4.7401 | 4.7506 |
| CGRRF1 | 0.009856 | 0.442311062 | 4.9988 | 4.7667 | 4.8351 | 4.6849 | 4.601 | 5.9061 | 6.0298 | 5.9266 |
| SRSF2 | 0.043386 | 0.434804934 | 8.1012 | 7.8181 | 7.919 | 7.9378 | 7.6991 | 8.6324 | 9.3176 | 9.3398 |
| KTI12 | 0.042724 | 0.434278804 | 4.4303 | 5.0237 | 4.861 | 4.8962 | 4.4716 | 5.9969 | 5.6723 | 6.1504 |
| DDIT3 | 0.018105 | 0.43415233 | 5.7348 | 5.501 | 5.2369 | 5.4976 | 5.2909 | 6.4984 | 6.665 | 6.8045 |
| RN45S | 0.02787 | 0.433667157 | 8.1241 | 8.1492 | 7.983 | 8.1983 | 7.6312 | 9.2015 | 9.0343 | 9.4317 |
| MAP4K3 | 0.041006 | 0.433372847 | 0.619 | 1.3791 | 0.93137 | 1.1586 | 1.1855 | 2.1032 | 2.4276 | 2.2523 |
| EPC1 | 0.0207 | 0.432350478 | 4.1722 | 4.2545 | 3.9589 | 4.1713 | 3.7618 | 5.3642 | 5.4004 | 5.0558 |
| DDX6 | 0.018088 | 0.429578431 | 3.9941 | 3.8419 | 3.6689 | 3.6702 | 3.7412 | 5.184 | 5.1052 | 4.7176 |
| RNF126 | 0.022373 | 0.428674132 | 5.015 | 4.9064 | 5.1289 | 5.2734 | 5.4939 | 6.2968 | 6.5554 | 6.3045 |
| NECAP1 | 0.003103 | 0.428044665 | 4.696 | 4.8764 | 4.8586 | 4.7728 | 4.8357 | 6.0896 | 6.1197 | 5.8869 |
| OTUD1 | 0.033764 | 0.425998373 | 3.6317 | 4.332 | 3.9094 | 4.244 | 4.059 | 5.1219 | 5.2659 | 5.4111 |
| NDNL2 | 0.026809 | 0.425783953 | 6.6915 | 6.7679 | 6.5738 | 7.0002 | 6.6589 | 7.7446 | 7.8489 | 8.3173 |
| RBBP6 | 0.011301 | 0.41852914 | 4.8925 | 4.842 | 4.5292 | 4.5793 | 4.7355 | 5.8432 | 6.0766 | 5.9971 |
| ELF2 | 0.043108 | 0.417775566 | 4.3748 | 4.4015 | 4.4571 | 4.3428 | 3.6778 | 5.3927 | 5.6484 | 5.4889 |
| C5orf41 | 0.02787 | 0.415949424 | 5.1932 | 4.934 | 4.8655 | 5.3169 | 5.2623 | 6.6632 | 6.3745 | 6.102 |
| LOC100129196 | 0.019175 | 0.413232814 | 2.0683 | 2.1184 | 2.4575 | 2.592 | 2.0951 | 3.4665 | 3.6058 | 3.5514 |
| BNIP1 | 0.032169 | 0.409136225 | 3.5523 | 3.5269 | 3.6969 | 4.0089 | 4.2036 | 5.0352 | 5.1932 | 5.0328 |
| ZNF136 | 0.040834 | 0.407432575 | 4.1023 | 3.5937 | 4.0757 | 3.5445 | 3.4628 | 5.0905 | 4.8484 | 5.2146 |
| C6orf226 | 0.016622 | 0.402713782 | 4.8518 | 5.155 | 5.0876 | 5.1157 | 4.7247 | 6.1551 | 6.2454 | 6.4969 |
| CCNL1 | 0.018088 | 0.400518211 | 6.8551 | 7.0557 | 6.65 | 7.2285 | 6.9034 | 8.1024 | 8.3374 | 8.336 |
| USP30 | 0.044723 | 0.399204527 | 1.8177 | 2.454 | 2.1219 | 1.8328 | 1.7696 | 3.0162 | 3.6147 | 3.3411 |
| RLF | 0.014482 | 0.398067929 | 3.3921 | 3.1763 | 3.1371 | 3.0795 | 3.3846 | 4.7343 | 4.6515 | 4.3027 |
| PET117 | 0.045075 | 0.397008011 | 4.3699 | 4.3303 | 4.8307 | 4.7594 | 4.1639 | 6.1617 | 5.7543 | 5.5548 |
| GRPEL1 | 0.026951 | 0.396826413 | 5.338 | 6.06 | 5.592 | 5.7297 | 5.3892 | 6.8907 | 6.9944 | 6.9805 |
| KBTBD2 | 0.012792 | 0.395665527 | 4.5224 | 4.4194 | 4.4614 | 4.6724 | 4.468 | 5.6809 | 6.1438 | 5.7144 |
| CLP1 | 0.020073 | 0.395164878 | 3.481 | 3.0647 | 3.3591 | 3.44 | 3.7615 | 4.8751 | 4.6361 | 4.771 |
| COQ10B | 0.018068 | 0.394245611 | 5.1821 | 5.0482 | 5.3116 | 5.42 | 5.6161 | 6.5296 | 6.8087 | 6.637 |
| LOC152217 | 0.014405 | 0.39327941 | 6.0298 | 6.2068 | 5.9363 | 6.2337 | 5.8472 | 7.242 | 7.3298 | 7.6196 |
| HAUS3 | 0.018088 | 0.390542954 | 4.4717 | 4.0769 | 4.2687 | 4.3183 | 3.8875 | 5.4221 | 5.578 | 5.6831 |
| EAPP | 0.013987 | 0.388785569 | 6.1477 | 5.7213 | 5.8505 | 6.2121 | 6.1143 | 7.3836 | 7.4308 | 7.302 |
| RSL24D1 | 0.037912 | 0.387367073 | 6.4892 | 6.8401 | 6.2804 | 6.6868 | 6.0812 | 7.5748 | 7.9684 | 7.9881 |
| ETF1 | 0.042724 | 0.386889457 | 4.1636 | 4.7359 | 4.0784 | 4.0139 | 4.0765 | 5.2819 | 5.8682 | 5.6009 |
| IKZF5 | 0.037568 | 0.385898458 | 4.126 | 4.694 | 4.1734 | 4.7564 | 4.0945 | 5.9145 | 5.7099 | 5.6033 |
| METTL21D | 0.011404 | 0.385821842 | 4.1 | 3.713 | 3.9102 | 3.7613 | 3.6967 | 5.2766 | 5.0436 | 5.3105 |
| MOAP1 | 0.005572 | 0.385283759 | 6.0601 | 5.9599 | 5.8643 | 6.1413 | 5.8582 | 7.447 | 7.2168 | 7.3945 |
| BCAS2 | 0.034628 | 0.384643373 | 6.1587 | 6.8589 | 6.1441 | 6.3534 | 6.0527 | 7.5692 | 7.8124 | 7.6943 |
| RHBDD2 | 0.018816 | 0.384431884 | 5.9461 | 5.5808 | 5.7429 | 5.8272 | 5.6885 | 7.0423 | 7.4912 | 6.8754 |
| C1orf55 | 0.023812 | 0.383573049 | 3.1065 | 2.8173 | 2.5206 | 3.1341 | 3.0727 | 4.5007 | 4.1522 | 4.2851 |
| H3F3B | 0.042724 | 0.380025198 | 9.3774 | 9.6794 | 8.8511 | 9.4448 | 9.4248 | 10.412 | 10.951 | 10.891 |
| CHD1 | 0.018068 | 0.377892179 | 4.0758 | 3.9806 | 4.1725 | 4.2398 | 4.2947 | 5.4938 | 5.8871 | 5.289 |
| DCTN6 | 0.039721 | 0.376852848 | 4.1759 | 4.0889 | 4.1057 | 4.4848 | 3.5174 | 5.3601 | 5.477 | 5.6103 |
| EIF1B | 0.023115 | 0.374864178 | 6.0726 | 6.705 | 6.1779 | 6.318 | 6.2137 | 7.8637 | 7.4451 | 7.8302 |
| DNAJB9 | 0.015597 | 0.37156162 | 5.1073 | 5.4292 | 5.061 | 5.4136 | 5.2926 | 6.5072 | 6.6008 | 6.9592 |
| MSL2 | 0.039858 | 0.365740732 | 4.1834 | 4.114 | 4.3824 | 4.5636 | 3.6374 | 5.5805 | 5.8325 | 5.4688 |
| EXOSC6 | 0.042051 | 0.365671472 | 4.0558 | 3.8066 | 4.0718 | 3.8219 | 3.7255 | 4.7815 | 5.6278 | 5.6338 |
| TOE1 | 0.022325 | 0.365549816 | 3.1413 | 3.1347 | 3.6791 | 3.5503 | 3.0308 | 4.6438 | 4.7623 | 4.8712 |
| CCDC59 | 0.018741 | 0.364883131 | 5.0225 | 5.1939 | 4.5975 | 5.1284 | 5.2804 | 6.4109 | 6.4836 | 6.6026 |
| SC5DL | 0.032031 | 0.36191092 | 1.9324 | 1.7665 | 2.3469 | 2.0045 | 2.0729 | 3.0494 | 3.7212 | 3.7022 |
| VPS37B | 0.039721 | 0.35735585 | 4.7795 | 4.1819 | 4.6283 | 4.6154 | 4.9279 | 5.8499 | 6.5302 | 5.9534 |
| RNF139 | 0.032102 | 0.355900661 | 5.9788 | 6.1026 | 5.2858 | 6.0629 | 5.6793 | 7.2227 | 7.2831 | 7.4312 |
| SPTY2D1 | 0.018105 | 0.355181013 | 2.4981 | 2.5615 | 2.541 | 2.8214 | 3.0118 | 4.1167 | 4.4468 | 3.9769 |
| ALG13 | 0.018088 | 0.352030746 | 3.6144 | 3.7716 | 3.5893 | 3.8834 | 3.1925 | 5.0374 | 5.091 | 5.221 |
| TOMM20 | 0.025995 | 0.351143629 | 4.7753 | 4.9302 | 5.1706 | 4.9652 | 4.3332 | 6.4629 | 6.2305 | 6.3409 |
| ZNF273 | 0.037397 | 0.351031389 | 1.5414 | 0.98076 | 1.9334 | 1.5915 | 1.2198 | 3.0864 | 2.9852 | 2.8195 |
| RBM3 | 0.033375 | 0.350270152 | 5.5662 | 6.083 | 5.3636 | 5.3344 | 5.531 | 6.7637 | 7.2065 | 7.2971 |
| KANSL2 | 0.01088 | 0.349970868 | 5.0633 | 5.3811 | 5.1186 | 5.3148 | 5.4134 | 6.5969 | 6.7295 | 6.9924 |
| KLHL15 | 0.037413 | 0.349857643 | 1.2045 | 2.1332 | 1.4177 | 1.8338 | 1.8095 | 3.0684 | 3.3432 | 3.1731 |
| CIRBP | 0.018068 | 0.345767213 | 7.1853 | 6.8966 | 6.7055 | 7.2682 | 7.2611 | 8.4326 | 8.6351 | 8.7187 |
| IRF2BP2 | 0.032448 | 0.344658597 | 4.7541 | 4.5839 | 4.2509 | 4.9554 | 4.4309 | 5.8398 | 6.0499 | 6.5057 |
| SIAH2 | 0.027636 | 0.341497434 | 4.5573 | 4.7795 | 5.152 | 4.7184 | 4.9837 | 5.9925 | 6.4774 | 6.6948 |
| EIF1 | 0.042724 | 0.339762792 | 10.269 | 10.536 | 9.7392 | 10.451 | 9.9228 | 11.371 | 11.88 | 11.972 |
| ADNP2 | 0.039721 | 0.333554279 | 2.484 | 2.5064 | 1.9638 | 2.3846 | 2.1765 | 4.0641 | 4.2471 | 3.35 |
| CTRL | 0.045497 | 0.330122518 | 2.5048 | 1.5822 | 2.0438 | 2.2182 | 2.4787 | 4.1001 | 3.7028 | 3.4905 |
| ANKRD37 | 0.019538 | 0.329375841 | 1.7823 | 2.0413 | 1.805 | 2.3572 | 2.2314 | 3.8072 | 3.3506 | 3.7791 |
| CNOT6L | 0.028889 | 0.329366696 | 3.7938 | 3.4424 | 3.9104 | 3.8735 | 4.0259 | 5.1089 | 5.8753 | 5.2501 |
| HNRNPA0 | 0.035877 | 0.328623249 | 5.0242 | 4.9307 | 4.8311 | 4.1604 | 4.3133 | 6.3738 | 6.155 | 6.2435 |
| JMJD6 | 0.038224 | 0.328101357 | 5.4652 | 4.9478 | 5.2083 | 5.0892 | 5.1474 | 6.1905 | 7.1147 | 7.0329 |
| PGRMC2 | 0.01774 | 0.325231767 | 3.2778 | 3.4727 | 3.7567 | 3.5444 | 3.2216 | 4.7732 | 5.2492 | 5.2029 |
| RNMT | 0.043022 | 0.324928262 | 2.6143 | 2.5217 | 2.7899 | 3.2512 | 2.2777 | 4.497 | 4.5086 | 3.9327 |
| ZNF639 | 0.018816 | 0.323932818 | 4.3642 | 4.2138 | 3.8314 | 4.5798 | 4.5063 | 5.9246 | 5.7968 | 6.0546 |
| PTP4A1 | 0.04566 | 0.322767368 | 5.3118 | 6.0151 | 4.9457 | 5.6279 | 5.118 | 6.8685 | 6.9891 | 7.2478 |
| DBF4 | 0.018088 | 0.320392716 | 3.1347 | 3.0216 | 2.7722 | 3.3332 | 2.8102 | 4.4749 | 4.9741 | 4.5204 |
| ISCA1 | 0.018539 | 0.317772643 | 5.1251 | 5.0816 | 5.3267 | 5.133 | 5.5751 | 6.9304 | 7.22 | 6.5563 |
| RNF138 | 0.023942 | 0.312251394 | 4.7467 | 5.0214 | 4.7409 | 5.3184 | 4.483 | 6.3518 | 6.4959 | 6.7762 |
| DYNLT1 | 0.046744 | 0.307978166 | 6.7174 | 7.251 | 6.8416 | 6.3232 | 6.8673 | 7.9862 | 8.6145 | 8.8969 |
| ARID5A | 0.018105 | 0.305001147 | 5.2322 | 4.9971 | 5.6056 | 5.4986 | 5.6666 | 6.8619 | 7.2814 | 7.1961 |
| LOC255512 | 0.03129 | 0.302407673 | 2.2532 | 1.3192 | 1.4383 | 1.4193 | 1.4635 | 3.2497 | 3.156 | 3.5067 |
| SETD1B | 0.037568 | 0.300539531 | 1.2857 | 1.3987 | 1.8974 | 1.9253 | 1.3122 | 3.783 | 3.0491 | 3.0626 |
| SELK | 0.03621 | 0.295612701 | 6.7382 | 7.0197 | 6.5061 | 6.5621 | 7.0368 | 7.9503 | 8.7639 | 8.8782 |
| JMY | 0.0226 | 0.293346944 | 1.8959 | 1.7143 | 2.5377 | 2.4203 | 1.9447 | 3.8348 | 4.0031 | 3.7778 |
| SLC2A3 | 0.027794 | 0.292569875 | 5.9722 | 6.3836 | 5.4215 | 6.0569 | 5.8844 | 7.4159 | 7.9229 | 7.8118 |
| JOSD1 | 0.021855 | 0.291352957 | 3.3972 | 3.0072 | 3.22 | 2.8982 | 2.6766 | 4.6135 | 5.1931 | 4.6504 |
| EPB41L4A-AS1 | 0.042724 | 0.285590485 | 3.4539 | 2.9784 | 3.7338 | 3.4634 | 2.8016 | 4.8007 | 4.9245 | 5.5574 |
| SBDSP1 | 0.009594 | 0.267344731 | 5.9338 | 5.6805 | 5.8001 | 5.908 | 5.3198 | 7.5897 | 7.6507 | 7.6546 |
| CDKN1B | 0.037413 | 0.263267209 | 2.7914 | 2.7171 | 3.536 | 2.5865 | 3.0455 | 5.3294 | 4.4871 | 4.7656 |
| CBX4 | 0.012792 | 0.25677915 | 3.6323 | 3.4947 | 3.4185 | 3.3893 | 4.0192 | 5.4766 | 5.8113 | 5.3687 |
| LOC100507217 | 0.007643 | 0.253692556 | 6.566 | 6.2869 | 6.3036 | 6.7518 | 6.3858 | 8.3098 | 8.3442 | 8.659 |
| BTG3 | 0.020073 | 0.250647765 | 3.7008 | 3.4682 | 3.5769 | 3.7893 | 3.7228 | 5.0387 | 5.9234 | 5.9815 |
| ZFP36L2 | 0.018088 | 0.24977023 | 8.284 | 8.2199 | 8.162 | 8.4193 | 8.156 | 9.8107 | 10.788 | 10.15 |
| ZNF295 | 0.014482 | 0.240301901 | 1.3717 | 1.1123 | 1.7622 | 1.5433 | 1.1456 | 3.5376 | 3.6615 | 3.1332 |
| TIPARP | 0.030628 | 0.234085349 | 4.1624 | 3.7201 | 3.0204 | 3.2106 | 3.2352 | 5.4818 | 5.4239 | 5.7882 |
| FBXO33 | 0.007643 | 0.230966121 | 3.8689 | 3.5752 | 3.9786 | 3.8973 | 3.5041 | 5.9898 | 5.6339 | 6.0135 |
| SBDS | 0.029854 | 0.230474643 | 6.9565 | 7.1539 | 6.148 | 7.2413 | 6.4457 | 8.7432 | 8.9099 | 9.0661 |
| SNHG3 | 0.027122 | 0.228857261 | 4.0585 | 5.0255 | 4.2503 | 4.4366 | 3.9102 | 6.5255 | 6.1815 | 6.6841 |
| MBOAT4 | 0.019607 | 0.222811549 | 1.7108 | 1.8445 | 1.361 | 1.5364 | 0.78828 | 3.6141 | 3.7573 | 3.4715 |
| ZC3H12A | 0.018816 | 0.22200232 | 4.2442 | 3.5261 | 3.7188 | 3.9066 | 4.2407 | 5.6631 | 6.3706 | 6.2622 |
| C20orf111 | 0.007749 | 0.218194497 | 4.8662 | 4.6903 | 4.7046 | 5.0768 | 5.0907 | 6.8159 | 7.0754 | 7.3548 |
| SLC45A1 | 0.032448 | 0.21111516 | -3.1315 | -2.229 | -3.0411 | -2.865 | -2.619 | -1.0203 | -0.64458 | 0.065214 |
| MTFP1 | 0.018088 | 0.203850053 | 4.1505 | 5.0967 | 4.2278 | 4.6501 | 4.6618 | 6.5686 | 6.9598 | 7.027 |
| TMEM2 | 0.044339 | 0.202297918 | 2.5063 | 3.3151 | 2.4955 | 2.5836 | 2.9741 | 4.4563 | 5.8654 | 4.9194 |
| ZNF394 | 0.007643 | 0.20119043 | 5.6067 | 5.2226 | 4.9266 | 5.4945 | 5.3631 | 7.4959 | 7.7544 | 7.6579 |
| IDI2-AS1 | 0.04588 | 0.173401293 | 4.3255 | 3.877 | 2.9462 | 4.1463 | 2.8581 | 5.9869 | 6.2952 | 6.1932 |
| RBM38 | 0.009856 | 0.153711059 | 5.7524 | 5.8694 | 6.2111 | 6.145 | 6.0929 | 8.213 | 8.9273 | 9.0073 |
| PFKFB3 | 0.029854 | 0.120380487 | 2.1649 | 2.945 | 1.3318 | 1.6524 | 1.8816 | 4.4981 | 5.3958 | 5.2545 |
| LZTS1 | 0.00402 | 0.105433118 | -6.6439 | -6.6439 | -6.6439 | -6.6439 | -6.6439 | -3.2778 | -3.9073 | -3.0098 |
| L1TD1 | 0.002206 | 0.092838487 | -6.6439 | -6.6439 | -6.6439 | -6.6439 | -6.6439 | -2.7625 | -3.3904 | -3.4914 |
| MAFF | 0.041988 | 0.08837602 | 2.6916 | 2.5976 | 0.78196 | 1.9016 | 1.2629 | 4.7163 | 5.7451 | 5.5806 |
| PDE4D | 0.016954 | 0.088239001 | 1.7773 | 2.0835 | 1.9696 | 2.6714 | 1.4525 | 4.8875 | 6.0427 | 5.5497 |
| DUSP2 | 0.030612 | 0.084004655 | 6.7585 | 7.72 | 6.1392 | 7.1357 | 6.7205 | 9.5425 | 10.713 | 11.149 |
| CREM | 0.012333 | 0.083588381 | 3.9017 | 3.5565 | 3.8307 | 3.1285 | 3.905 | 6.6283 | 7.8267 | 7.2801 |
| ZNF331 | 0.015597 | 0.081089168 | 3.3422 | 3.279 | 3.7536 | 4.0037 | 2.5776 | 6.547 | 7.1886 | 7.3111 |
| NPHS1 | 0.021855 | 0.076742345 | -6.6439 | -6.6439 | -6.6439 | -6.6439 | -6.6439 | -3.1532 | -1.7824 | -3.8846 |
| PER1 | 0.019538 | 0.074973911 | 2.3045 | 1.5696 | 1.311 | 2.5548 | 0.97943 | 5.3996 | 5.9301 | 5.1143 |
| OVOL1 | 1.90E-05 | 0.044789364 | -6.6439 | -6.6439 | -6.6439 | -6.6439 | -6.6439 | -2.3792 | -2.0055 | -2.1049 |
| HYAL4 | 0.001249 | 0.044044327 | -6.6439 | -6.6439 | -6.6439 | -6.6439 | -6.6439 | -2.0236 | -2.6479 | -1.7455 |
| DNALI1 | 0.006965 | 0.042744026 | -6.6439 | -6.6439 | -6.6439 | -6.6439 | -6.6439 | -2.1745 | -2.7997 | -1.3131 |
| LOC147670 | 0.006711 | 0.040864732 | -6.6439 | -6.6439 | -6.6439 | -6.6439 | -6.6439 | -2.11 | -2.7348 | -1.2479 |
| RIPK4 | 0.019046 | 0.040460936 | -6.6439 | -6.6439 | -6.6439 | -6.6439 | -6.6439 | -1.7593 | -3.3872 | -0.90322 |
| GALNT9 | 0.000388 | 0.038288807 | -6.6439 | -6.6439 | -6.6439 | -6.6439 | -6.6439 | -1.4922 | -2.1125 | -2.2062 |
| CNIH2 | 0.000249 | 0.028230525 | -6.6439 | -6.6439 | -6.6439 | -6.6439 | -6.6439 | -1.0571 | -1.6727 | -1.7621 |
| UPK3B | 0.015597 | 0.02298447 | -6.6439 | -6.6439 | -6.6439 | -6.6439 | -6.6439 | 0.29889 | -1.9046 | -1.9964 |
| DHRS4L1 | 0.013915 | 0.018150633 | -6.6439 | -6.6439 | -6.6439 | -6.6439 | -6.6439 | 0.63541 | -1.5638 | -1.6518 |
| ACHE | 5.60E-07 | 0.012867302 | -6.6439 | -6.6439 | -6.6439 | -6.6439 | -6.6439 | -0.29992 | -0.50828 | -0.28306 |
| LOC100506274 | 0.002288 | 0.012423937 | -6.6439 | -6.6439 | -6.6439 | -6.6439 | -6.6439 | -0.41123 | -1.0168 | 0.48853 |
| HCRT | 4.28E-05 | 0.009153868 | -6.6439 | -6.6439 | -6.6439 | -6.6439 | -6.6439 | 0.53209 | -0.04766 | -0.10192 |
| RBM24 | 0.037883 | 0.009006817 | -6.6439 | -6.6439 | -6.6439 | -6.6439 | -6.6439 | 1.7903 | -2.6418 | 1.3041 |
| ADRA2B | 0.000249 | 0.004044685 | -6.6439 | -6.6439 | -6.6439 | -6.6439 | -6.6439 | 1.408 | 1.8284 | 0.68117 |
| CYCSP52 | 5.40E-06 | 0.002570502 | -6.6439 | -6.6439 | -6.6439 | -6.6439 | -6.6439 | 2.2843 | 1.7809 | 1.8143 |
