# Supplementary material for: Transcriptional profiling demonstrates altered characteristics of CD8 + cytotoxic T‐cells and regulatory T‐cells in TP53‐mutated acute myeloid leukemia
Source: Cancer Med. 2022 Mar 16;11(15):3023–32. doi: 10.1002/cam4.4661 (PMC9359873; doi:10.1002/cam4.4661)
Supplement: Supplementary file 4 — TableS 3 [file CAM4-11-3023-s001.docx]

**Supporting table 3**

Differentially expressed genes by AML Treg vs. control Treg.

| **Gene name** | **FDR<0.05** | **Fold change** | **Expression levels (log2 FPKM values)** | | | | | | |
| --- | --- | --- | --- | --- | --- | --- | --- | --- | --- |
|  |  |  | **AML-A** | **AML-B** | **AML-C** | **AML-D** | **Normal-A** | **Normal-B** | **Normal-C** |
| LIN7A | 0.04998 | 84.00435155 | 0.65857 | -1.5268 | 1.1231 | -1.2609 | -6.6439 | -6.6439 | -6.6439 |
| NIPSNAP3B | 0.031593 | 78.10858962 | -1.2848 | -0.15452 | 0.90797 | -0.89461 | -6.6439 | -6.6439 | -6.6439 |
| LOC100507392 | 0.004712 | 74.17428618 | -0.76116 | -0.62575 | -0.56129 | 0.22399 | -6.6439 | -6.6439 | -6.6439 |
| LOC100506668 | 0.042193 | 64.0226379 | 0.38867 | -0.28941 | -2.2273 | -0.44552 | -6.6439 | -6.6439 | -6.6439 |
| LRRC69 | 0.024652 | 60.88732677 | -1.7955 | -0.6605 | -0.59619 | 0.18887 | -6.6439 | -6.6439 | -6.6439 |
| GPR77 | 0.036667 | 60.2406473 | -0.74578 | 0.71174 | -1.5457 | -1.3452 | -6.6439 | -6.6439 | -6.6439 |
| KCTD15 | 0.049252 | 39.10665957 | -1.9993 | -0.46084 | -0.40099 | -2.5571 | -6.6439 | -6.6439 | -6.6439 |
| RIMBP3 | 0.000173 | 37.06085904 | -1.5911 | -1.2468 | -1.41 | -1.4804 | -6.6439 | -6.6439 | -6.6439 |
| CISH | 0.016021 | 34.8110933 | 7.4319 | 6.9222 | 6.6299 | 7.5923 | 2.4609 | 1.5174 | 2.0895 |
| CXorf21 | 0.04807 | 28.81979276 | 3.1401 | 3.7066 | 4.5664 | 2.6434 | -0.63969 | -1.7951 | -1.5698 |
| CD101 | 0.042549 | 13.76506875 | 2.2732 | 2.6802 | 2.0609 | 3.5053 | -1.1805 | -0.75822 | -1.5204 |
| FSD1L | 0.011674 | 12.83378657 | 1.2722 | 1.3137 | 1.2883 | 1.5825 | -2.7778 | -1.9329 | -2.2424 |
| TNFAIP8L2 | 0.036416 | 11.34335013 | 5.8256 | 5.6933 | 6.2557 | 5.9081 | 3.1447 | 1.8393 | 2.2667 |
| TREML2 | 0.037069 | 9.329770374 | 1.2405 | 1.9312 | 2.161 | 1.1108 | -1.6891 | -1.8424 | -1.3014 |
| PAQR8 | 0.043689 | 7.537782799 | 4.146 | 3.2852 | 4.5545 | 3.7523 | 0.86548 | 1.1277 | 1.0679 |
| CCR5 | 0.048626 | 7.064087606 | 3.5121 | 3.0935 | 2.9837 | 2.7887 | 0.7748 | -0.37873 | 0.42592 |
| FAM129B | 0.037858 | 6.119531477 | 0.80668 | 0.067818 | 0.25329 | 0.56243 | -2.105 | -1.8433 | -2.6243 |
| C9orf64 | 0.04998 | 6.021988703 | 2.1906 | 2.9452 | 2.0887 | 2.6165 | 0.13083 | 0.12841 | -0.64921 |
| LOC100505812 | 0.044971 | 5.98779394 | 4.3056 | 4.4733 | 4.2069 | 4.3711 | 1.2372 | 1.6344 | 2.4 |
| PLEKHO2 | 0.006975 | 5.884472677 | 3.408 | 3.6204 | 3.5672 | 3.3868 | 0.9741 | 0.69516 | 1.1468 |
| FUT7 | 0.038133 | 5.794663763 | 4.9926 | 4.8193 | 4.0025 | 4.4497 | 1.906 | 2.1665 | 2.0214 |
| CEP19 | 0.036829 | 5.780908562 | 2.3663 | 2.4483 | 2.948 | 2.2752 | 0.34612 | 0.021281 | -0.43294 |
| C6orf211 | 0.036667 | 5.748126425 | 3.8645 | 4.5312 | 3.8093 | 4.1235 | 1.9434 | 1.4451 | 1.2886 |
| PLSCR1 | 0.041834 | 4.832348921 | 4.8524 | 4.7325 | 4.8216 | 4.0888 | 2.6314 | 2.3067 | 2.1152 |
| ZNF613 | 0.002183 | 4.260850587 | 2.9643 | 2.7557 | 2.9274 | 2.7901 | 0.74961 | 0.777 | 0.77809 |
| MED18 | 0.046159 | 3.771669684 | 3.3341 | 3.1619 | 3.0832 | 3.4866 | 1.5472 | 0.88894 | 1.6176 |
| RAB10 | 0.011304 | 3.667147932 | 6.0195 | 6.1177 | 6.2983 | 5.9042 | 4.1642 | 4.1467 | 4.3199 |
| DOLPP1 | 0.036667 | 3.646237027 | 2.8062 | 3.5043 | 3.067 | 2.8688 | 1.1644 | 1.2773 | 1.1438 |
| TMEM140 | 0.032548 | 3.627728495 | 4.5254 | 4.487 | 4.3405 | 4.2987 | 2.576 | 2.8729 | 2.2126 |
| UNC93B1 | 0.042193 | 3.624838184 | 4.6794 | 4.4247 | 4.9572 | 4.1917 | 2.738 | 2.8178 | 2.5602 |
| C14orf119 | 0.006975 | 3.612797799 | 5.343 | 5.2116 | 5.2378 | 5.2474 | 3.2106 | 3.5555 | 3.4544 |
| CALHM2 | 0.011674 | 3.612589137 | 5.5499 | 5.3602 | 5.4648 | 5.5599 | 3.8613 | 3.5198 | 3.5109 |
| PIM1 | 0.046159 | 3.592051964 | 7.5746 | 7.7487 | 7.1143 | 7.2143 | 5.7333 | 5.2854 | 5.6858 |
| METTL13 | 0.03403 | 3.529486361 | 3.8627 | 3.9815 | 4.0622 | 4.1661 | 2.4843 | 2.2621 | 1.8496 |
| APOBEC3F | 0.01728 | 3.436186472 | 3.4447 | 3.3531 | 3.2226 | 3.1099 | 1.5765 | 1.6419 | 1.2869 |
| SH2D3C | 0.024652 | 3.415842541 | 4.8106 | 4.4938 | 4.3396 | 4.7419 | 2.9421 | 2.6831 | 2.8475 |
| MCAT | 0.046159 | 3.376484125 | 2.5093 | 2.851 | 3.1234 | 2.45 | 0.89411 | 0.864 | 1.1756 |
| TMEM102 | 0.036667 | 3.353826933 | 3.4466 | 3.1365 | 3.4345 | 3.5755 | 1.907 | 1.3367 | 1.7137 |
| COPB2 | 0.030505 | 3.270531227 | 5.2843 | 5.1519 | 5.4675 | 5.356 | 3.8692 | 3.6121 | 3.3349 |
| TMEM165 | 0.036332 | 3.206983024 | 5.471 | 5.9088 | 5.7632 | 5.3108 | 3.8678 | 3.9012 | 4.0277 |
| TXNDC15 | 0.030984 | 3.118783362 | 2.9738 | 3.1299 | 2.8879 | 3.1206 | 1.1973 | 1.2734 | 1.6905 |
| TRIM47 | 0.049252 | 3.111406053 | 3.018 | 3.1988 | 2.8364 | 3.1008 | 1.2717 | 1.1169 | 1.8142 |
| SIT1 | 0.032548 | 3.024524146 | 7.89 | 7.724 | 7.362 | 7.4443 | 6.019 | 6.0565 | 5.9496 |
| LPAR5 | 0.048626 | 3.022777993 | 3.4559 | 3.1518 | 3.3446 | 3.5596 | 1.8647 | 2.065 | 1.4166 |
| CCT5 | 0.049252 | 3.006722551 | 6.1636 | 6.5683 | 6.3142 | 5.8856 | 4.4232 | 4.7527 | 4.7583 |
| MOB3A | 0.013717 | 2.943042864 | 6.1613 | 6.0169 | 6.1618 | 6.1395 | 4.4031 | 4.5126 | 4.772 |
| ANXA4 | 0.03403 | 2.69826735 | 4.0805 | 3.9509 | 3.9445 | 4.0693 | 2.2771 | 2.6785 | 2.7822 |
| SNAPIN | 0.042193 | 2.656358629 | 6.3749 | 6.6597 | 6.4307 | 6.1701 | 4.7801 | 5.1934 | 5.0247 |
| TRIM25 | 0.036332 | 2.572982609 | 3.6312 | 3.1681 | 3.4855 | 3.5415 | 2.1607 | 1.9425 | 2.1762 |
| HDAC8 | 0.011674 | 2.538043403 | 4.1721 | 4.4984 | 4.307 | 4.3775 | 2.9766 | 2.9768 | 3.0317 |
| AHSA1 | 0.04934 | 2.502710977 | 6.0577 | 6.5182 | 6.2843 | 6.2355 | 4.9465 | 4.7113 | 5.1935 |
| CETN3 | 0.004712 | 2.464764648 | 4.5723 | 4.6357 | 4.7299 | 4.6059 | 3.3282 | 3.2673 | 3.408 |
| TM9SF1 | 0.042131 | 2.459289366 | 4.4008 | 4.3542 | 3.9724 | 4.1677 | 2.739 | 3.0778 | 2.9598 |
| TP53RK | 0.036667 | 2.404785543 | 3.2832 | 3.422 | 3.5387 | 3.7188 | 2.2842 | 2.3234 | 2.0667 |
| EIF2B2 | 0.016527 | 2.384274022 | 4.8541 | 4.8619 | 5.0732 | 4.8838 | 3.6832 | 3.7818 | 3.5291 |
| UMPS | 0.036829 | 2.383268687 | 2.7149 | 2.7849 | 2.9963 | 2.6134 | 1.6785 | 1.5635 | 1.3313 |
| LEO1 | 0.047627 | 2.365057161 | 4.5554 | 4.9545 | 4.4475 | 4.7609 | 3.525 | 3.4896 | 3.2985 |
| DUSP18 | 0.041834 | 2.283465594 | 4.1135 | 4.2859 | 4.0612 | 3.7711 | 2.8564 | 2.8193 | 2.9244 |
| PRKAG1 | 0.046159 | 2.265335627 | 4.4925 | 4.8568 | 4.7251 | 4.7429 | 3.5892 | 3.2886 | 3.696 |
| PYCR2 | 0.036332 | 2.238178199 | 5.208 | 5.133 | 5.3763 | 5.0472 | 4.1573 | 3.8426 | 4.0865 |
| SNX1 | 0.01923 | 2.235206713 | 3.7167 | 3.6131 | 3.6068 | 3.4749 | 2.5378 | 2.2908 | 2.4988 |
| C9orf23 | 0.042549 | 2.230911533 | 5.0235 | 5.1585 | 4.9137 | 5.0959 | 4.1472 | 3.6947 | 3.8289 |
| TMEM223 | 0.049252 | 2.220305792 | 5.5282 | 5.2482 | 5.6073 | 5.1316 | 4.1298 | 4.2526 | 4.3018 |
| ACAD9 | 0.043689 | 2.19769578 | 3.7387 | 3.9255 | 3.9183 | 3.8076 | 2.4666 | 2.9457 | 2.7223 |
| THOC3 | 0.031017 | 2.184003155 | 5.0185 | 5.2078 | 5.0253 | 4.8399 | 3.9698 | 3.9345 | 3.7834 |
| VTA1 | 0.048651 | 2.182767331 | 4.839 | 5.1568 | 5.0082 | 5.1813 | 3.6868 | 4.0273 | 4.0464 |
| ARFRP1 | 0.036667 | 2.18114144 | 4.5995 | 4.8203 | 4.6675 | 4.4941 | 3.6582 | 3.3337 | 3.5689 |
| MTIF3 | 0.024652 | 2.166938791 | 6.3434 | 6.2503 | 6.2422 | 6.2122 | 5.0178 | 5.3473 | 5.074 |
| SDF2 | 0.042128 | 2.164424195 | 6.0335 | 5.817 | 5.7246 | 5.6779 | 4.7792 | 4.5121 | 4.8065 |
| EXOSC2 | 0.03403 | 2.164074044 | 3.8001 | 4.0382 | 4.069 | 3.8957 | 3.0092 | 2.6968 | 2.805 |
| SNRNP40 | 0.042083 | 2.160776321 | 5.8046 | 6.266 | 5.9756 | 5.9784 | 4.8605 | 4.8249 | 4.9984 |
| TRUB2 | 0.049883 | 2.152741282 | 5.2047 | 5.1426 | 5.007 | 4.718 | 4.0144 | 3.8969 | 3.8244 |
| MGAT1 | 0.048626 | 2.151932969 | 6.0742 | 6.1804 | 6.0701 | 6.2717 | 5.3013 | 4.8093 | 5.0198 |
| RGP1 | 0.041834 | 2.13322082 | 2.4499 | 2.6736 | 2.6365 | 2.7252 | 1.5491 | 1.7151 | 1.3206 |
| TPRA1 | 0.037858 | 2.131816609 | 4.1093 | 4.2847 | 4.534 | 4.323 | 3.3381 | 3.1452 | 3.1787 |
| CPSF3 | 0.049051 | 2.110509815 | 4.6059 | 5.1004 | 4.9136 | 4.7766 | 3.7072 | 3.7411 | 3.8663 |
| YIPF3 | 0.01923 | 2.110278586 | 6.4002 | 6.4162 | 6.1734 | 6.2554 | 5.2383 | 5.3163 | 5.147 |
| PRKAR1A | 0.024652 | 2.061972289 | 6.4186 | 6.5713 | 6.7152 | 6.4366 | 5.5272 | 5.5145 | 5.4325 |
| DAPP1 | 0.017416 | 2.048640242 | 4.281 | 4.4643 | 4.4919 | 4.3392 | 3.2611 | 3.4443 | 3.3729 |
| TMCO1 | 0.029118 | 2.021965519 | 4.8605 | 5.1279 | 4.9245 | 4.944 | 3.97 | 3.8229 | 4.0525 |
| DCLRE1A | 0.03403 | 2.013329573 | 2.594 | 2.3418 | 2.284 | 2.5488 | 1.4737 | 1.3539 | 1.4701 |
| HNRNPF | 0.037858 | 2.009333071 | 7.6809 | 7.7199 | 7.3961 | 7.6077 | 6.7405 | 6.4961 | 6.5467 |
| HSPA8 | 0.048626 | 1.975109829 | 10.143 | 9.9054 | 9.7296 | 10.058 | 8.8718 | 9.0488 | 9.0106 |
| C7orf11 | 0.046159 | 1.966288418 | 6.0665 | 6.1912 | 6.4258 | 6.1352 | 5.074 | 5.3199 | 5.2937 |
| ASH2L | 0.036667 | 1.916722827 | 4.7586 | 4.5847 | 4.3989 | 4.6705 | 3.6523 | 3.6108 | 3.7305 |
| GLB1 | 0.037002 | 1.901065812 | 4.8691 | 4.7849 | 4.7433 | 5.0046 | 3.9525 | 3.7737 | 4.0448 |
| ERGIC2 | 0.036365 | 1.861565736 | 5.3777 | 5.3211 | 5.6048 | 5.3002 | 4.537 | 4.4243 | 4.552 |
| SPCS2 | 0.03403 | 1.846604694 | 5.4058 | 5.7411 | 5.5045 | 5.5113 | 4.6189 | 4.6752 | 4.6733 |
| DPY30 | 0.048225 | 1.806367928 | 6.6253 | 6.5428 | 6.6422 | 6.8706 | 5.6913 | 5.8341 | 5.926 |
| ANXA6 | 0.02464 | 1.791063735 | 6.5803 | 6.6798 | 6.6752 | 6.5553 | 5.7339 | 5.9137 | 5.6979 |
| HECTD3 | 0.030505 | 1.787095586 | 4.2476 | 4.264 | 4.0904 | 4.3102 | 3.4629 | 3.2776 | 3.4308 |
| ATP6V0E1 | 0.036416 | 1.772978564 | 8.6958 | 8.7004 | 8.4297 | 8.5804 | 7.7036 | 7.7779 | 7.8447 |
| CHORDC1 | 0.048225 | 1.743204227 | 4.537 | 4.3271 | 4.3405 | 4.4361 | 3.6213 | 3.4472 | 3.7568 |
| NMT1 | 0.032548 | 1.73467651 | 4.3958 | 4.3295 | 4.3092 | 4.1583 | 3.5992 | 3.4181 | 3.4933 |
| GEMIN7 | 0.004712 | 1.732293474 | 4.6329 | 4.7496 | 4.6327 | 4.6834 | 3.8973 | 3.868 | 3.8806 |
| FNTA | 0.04146 | 1.68276476 | 6.5061 | 6.6743 | 6.7208 | 6.8172 | 5.9192 | 5.9844 | 5.8827 |
| APEH | 0.041834 | 1.673845728 | 5.368 | 5.428 | 5.2819 | 5.3009 | 4.6349 | 4.4427 | 4.727 |
| NPRL2 | 0.03403 | 1.666918227 | 5.1428 | 5.0837 | 5.0735 | 5.1014 | 4.5198 | 4.3275 | 4.2422 |
| SEC23IP | 0.036416 | 1.654772985 | 3.2234 | 3.3579 | 3.2701 | 3.477 | 2.634 | 2.5326 | 2.6498 |
| RUFY1 | 0.049252 | 1.642573852 | 4.4807 | 4.6431 | 4.448 | 4.4983 | 3.747 | 3.967 | 3.6907 |
| SMARCD2 | 0.04998 | 1.624523706 | 6.073 | 6.0731 | 6.0517 | 6.1784 | 5.3936 | 5.5593 | 5.2292 |
| MECP2 | 0.031272 | 1.622113718 | 3.6975 | 3.4592 | 3.5508 | 3.6304 | 2.8628 | 2.8724 | 2.9246 |
| ARAP1 | 0.040973 | 1.573570381 | 4.3417 | 4.406 | 4.2316 | 4.3886 | 3.7712 | 3.728 | 3.5646 |
| RPS6KA1 | 0.042193 | 1.553637362 | 6.1997 | 6.316 | 6.2893 | 6.2132 | 5.6283 | 5.4875 | 5.7409 |
| HADHB | 0.04998 | 1.546215796 | 5.9451 | 6.1559 | 5.931 | 6.0203 | 5.2693 | 5.4353 | 5.4484 |
| CTR9 | 0.036009 | 1.512821362 | 4.7704 | 4.9053 | 4.7881 | 4.8889 | 4.2622 | 4.3157 | 4.1449 |
| ARL8B | 0.025374 | 1.383062879 | 5.108 | 5.1156 | 5.0758 | 5.0714 | 4.5968 | 4.5675 | 4.7102 |
| BIN2 | 0.042848 | 0.788327495 | 7.9036 | 8.0444 | 8.0338 | 8.003 | 8.3446 | 8.3294 | 8.344 |
| RBMX | 0.04807 | 0.752002007 | 7.4496 | 7.4737 | 7.3264 | 7.3554 | 7.8657 | 7.802 | 7.7697 |
| LSM14A | 0.037081 | 0.703038187 | 5.9028 | 5.93 | 5.8435 | 5.9848 | 6.3462 | 6.5095 | 6.4151 |
| PAIP2 | 0.030387 | 0.691064256 | 7.7785 | 7.8039 | 7.911 | 7.8791 | 8.3132 | 8.3832 | 8.4323 |
| URI1 | 0.048651 | 0.682854498 | 4.6739 | 4.4912 | 4.6553 | 4.5778 | 5.2278 | 5.0494 | 5.1725 |
| ITM2B | 0.041834 | 0.675510297 | 9.0303 | 8.8536 | 9.0456 | 9.0243 | 9.626 | 9.5327 | 9.5045 |
| AFF4 | 0.03403 | 0.644223578 | 2.8131 | 2.7468 | 2.9583 | 2.8438 | 3.4044 | 3.5247 | 3.4955 |
| TOMM7 | 0.046159 | 0.642658892 | 6.6274 | 6.7249 | 6.6728 | 6.5022 | 7.2492 | 7.3774 | 7.1825 |
| MCPH1 | 0.041834 | 0.641094299 | 4.043 | 4.0621 | 4.0463 | 4.0721 | 4.6575 | 4.8563 | 4.578 |
| CSDE1 | 0.044721 | 0.639105064 | 6.9389 | 6.6803 | 6.873 | 6.7503 | 7.5121 | 7.4396 | 7.4178 |
| TRAM1 | 0.048626 | 0.635013087 | 6.9416 | 6.6412 | 6.808 | 6.7437 | 7.4152 | 7.4025 | 7.4986 |
| RPL21P28 | 0.032548 | 0.615074526 | 11.338 | 11.242 | 11.307 | 11.115 | 11.887 | 11.977 | 11.991 |
| FOXJ3 | 0.042193 | 0.608276881 | 3.9123 | 3.778 | 3.9469 | 4.0504 | 4.5921 | 4.7411 | 4.5841 |
| MRPS30 | 0.037002 | 0.601717595 | 5.46 | 5.7136 | 5.543 | 5.4991 | 6.2004 | 6.3196 | 6.3403 |
| C5orf44 | 0.032989 | 0.601175572 | 4.0859 | 4.0873 | 4.083 | 4.0951 | 4.7349 | 4.9857 | 4.7453 |
| CCNK | 0.032548 | 0.597478335 | 2.8529 | 2.8028 | 2.9454 | 3.0558 | 3.6142 | 3.675 | 3.6826 |
| CCNI | 0.028527 | 0.589804351 | 7.1895 | 7.3301 | 7.2163 | 7.2248 | 7.9953 | 8.1168 | 7.8935 |
| RPLP2 | 0.049252 | 0.589034787 | 9.3564 | 9.4436 | 9.5017 | 9.3024 | 9.9808 | 10.253 | 10.26 |
| PAN3 | 0.036365 | 0.587753513 | 4.1238 | 4.2831 | 4.3694 | 4.1947 | 5.1055 | 4.9927 | 4.9302 |
| KRT10 | 0.048225 | 0.583346572 | 4.9889 | 5.0641 | 4.8284 | 4.7783 | 5.6382 | 5.6361 | 5.8032 |
| SF1 | 0.048626 | 0.578761832 | 6.0551 | 5.8725 | 5.8891 | 5.9736 | 6.8103 | 6.8512 | 6.5481 |
| CMPK1 | 0.012858 | 0.575375281 | 6.5102 | 6.6113 | 6.4873 | 6.4839 | 7.2388 | 7.3436 | 7.3794 |
| VAPA | 0.004712 | 0.571328242 | 4.8836 | 4.9385 | 4.9211 | 4.8661 | 5.6603 | 5.7576 | 5.7119 |
| KLHDC2 | 0.048626 | 0.559370086 | 6.0817 | 6.1112 | 5.9541 | 6.3201 | 7.0302 | 6.9828 | 6.8517 |
| C9orf78 | 0.032548 | 0.557444534 | 6.8499 | 6.8486 | 6.7726 | 6.6381 | 7.5024 | 7.6483 | 7.7105 |
| RPS25 | 0.031272 | 0.557096832 | 11.349 | 11.379 | 11.314 | 11.302 | 12.013 | 12.217 | 12.31 |
| BCL10 | 0.013717 | 0.552281228 | 3.2761 | 3.1668 | 3.327 | 3.3732 | 4.0972 | 4.1659 | 4.1638 |
| PCNP | 0.017272 | 0.542116574 | 6.5096 | 6.3745 | 6.4032 | 6.4898 | 7.3619 | 7.2143 | 7.4066 |
| RG9MTD1 | 0.048651 | 0.539660876 | 5.3731 | 5.5435 | 5.271 | 5.3585 | 6.1615 | 6.4747 | 6.193 |
| MED10 | 0.018246 | 0.517381393 | 6.7452 | 6.4804 | 6.6566 | 6.7002 | 7.5872 | 7.6193 | 7.5824 |
| ARID2 | 0.048626 | 0.514496569 | 2.9627 | 2.716 | 2.8696 | 3.1269 | 3.9273 | 3.7529 | 3.9525 |
| BCLAF1 | 0.046159 | 0.505012709 | 5.7146 | 5.3643 | 5.4307 | 5.7241 | 6.5921 | 6.5447 | 6.4953 |
| AIMP1 | 0.048626 | 0.503821023 | 4.8559 | 4.5266 | 4.555 | 4.9083 | 5.7062 | 5.7215 | 5.6737 |
| SVIP | 0.037081 | 0.498730812 | 4.9377 | 4.9381 | 4.6009 | 4.9413 | 5.8235 | 5.9273 | 5.8237 |
| PITHD1 | 0.03403 | 0.494685493 | 5.0366 | 5.1481 | 5.2985 | 5.399 | 6.2945 | 6.1945 | 6.2189 |
| BNIP2 | 0.02344 | 0.489489555 | 5.9665 | 6.139 | 6.0601 | 5.935 | 6.972 | 7.1987 | 6.9967 |
| DDA1 | 0.029118 | 0.487517211 | 2.4889 | 2.2902 | 2.4496 | 2.643 | 3.4719 | 3.4764 | 3.5649 |
| ATP6V1G1 | 0.01852 | 0.486234829 | 7.2572 | 7.1896 | 7.3816 | 7.2845 | 8.2612 | 8.2331 | 8.4612 |
| HSPA13 | 0.018679 | 0.466093615 | 3.081 | 2.9936 | 3.1924 | 2.9411 | 4.1809 | 4.2377 | 4.0414 |
| TWF1 | 0.038437 | 0.459220293 | 4.2983 | 3.9506 | 3.9998 | 4.0646 | 5.1626 | 5.0663 | 5.3743 |
| PNO1 | 0.02801 | 0.455135067 | 3.7945 | 3.8277 | 3.7658 | 3.9624 | 4.9594 | 5.1489 | 4.8114 |
| MBIP | 0.034222 | 0.453460929 | 4.2798 | 4.0602 | 3.9676 | 4.0702 | 5.0602 | 5.3801 | 5.2659 |
| RSL24D1 | 0.037081 | 0.452613155 | 6.3933 | 6.8052 | 6.4052 | 6.4373 | 7.641 | 7.6196 | 7.7011 |
| G3BP2 | 0.011674 | 0.449987929 | 5.5716 | 5.4324 | 5.6249 | 5.6372 | 6.7299 | 6.8003 | 6.6255 |
| SLC25A36 | 0.037858 | 0.449572311 | 3.9488 | 4.1898 | 3.9585 | 3.8958 | 5.1306 | 5.354 | 4.9702 |
| RBBP6 | 0.031017 | 0.438791753 | 4.571 | 4.6443 | 4.4958 | 4.8632 | 5.8774 | 5.9056 | 5.7129 |
| GRPEL1 | 0.048626 | 0.438216801 | 5.7257 | 5.6493 | 5.2396 | 5.572 | 6.806 | 6.8176 | 6.5872 |
| RBM7 | 0.049051 | 0.437372228 | 4.1637 | 4.3076 | 4.7092 | 4.5063 | 5.5976 | 5.6891 | 5.5576 |
| ZC3H7A | 0.043411 | 0.421630207 | 5.419 | 5.189 | 5.0073 | 5.4389 | 6.597 | 6.5854 | 6.3461 |
| CLP1 | 0.049556 | 0.421522986 | 4.0864 | 3.538 | 3.8412 | 3.6306 | 5.1037 | 5.056 | 4.9014 |
| FEM1B | 0.026922 | 0.414666974 | 3.8986 | 3.6604 | 3.6878 | 3.9653 | 5.2062 | 5.0381 | 4.9747 |
| HIAT1 | 0.04934 | 0.414114051 | 5.3099 | 4.9828 | 5.1841 | 5.6048 | 6.5842 | 6.4934 | 6.5493 |
| EIF1B | 0.024652 | 0.411279726 | 6.605 | 6.7468 | 6.4364 | 6.6247 | 7.9251 | 8.0074 | 7.7226 |
| FAM200B | 0.044994 | 0.398002677 | 2.1303 | 2.3248 | 2.6591 | 2.5304 | 3.615 | 3.7302 | 3.8757 |
| ELF1 | 0.036667 | 0.385488231 | 6.5492 | 6.2494 | 6.379 | 6.6749 | 7.7088 | 8.0425 | 7.7638 |
| SIAH2 | 0.008315 | 0.384261024 | 4.7117 | 4.7719 | 4.8253 | 4.87 | 6.1622 | 6.0511 | 6.3104 |
| FLJ10038 | 0.046356 | 0.382036733 | 2.7813 | 2.7996 | 2.9794 | 2.5199 | 3.8809 | 4.3122 | 4.2817 |
| LOC282997 | 0.049051 | 0.381798504 | 3.2882 | 2.9314 | 2.8412 | 2.6226 | 4.3214 | 4.3807 | 4.2278 |
| ALG13 | 0.046159 | 0.377315113 | 3.8979 | 3.7359 | 3.6462 | 3.8091 | 4.87 | 5.4904 | 5.1749 |
| BCL2L11 | 0.043689 | 0.373252838 | 3.1218 | 3.1359 | 3.2185 | 3.6515 | 4.6217 | 4.6418 | 4.8476 |
| MMD | 0.036667 | 0.361215949 | 2.7197 | 2.7614 | 2.8787 | 2.621 | 4.244 | 3.9221 | 4.4767 |
| KBTBD8 | 0.046159 | 0.318575741 | 1.8753 | 1.3993 | 1.6871 | 1.3806 | 3.1058 | 3.5379 | 3.0639 |
| ODZ1 | 0.049252 | 0.310085935 | -1.1963 | -1.0762 | -1.0177 | -1.674 | 0.64374 | 0.49171 | 0.20918 |
| SNHG3 | 0.043689 | 0.30730651 | 4.2947 | 4.7725 | 4.9317 | 4.3933 | 6.141 | 6.3672 | 6.3927 |
| HSF2 | 0.004595 | 0.294106052 | 3.672 | 3.5366 | 3.5911 | 3.7806 | 5.4362 | 5.3819 | 5.4139 |
| RBM38 | 0.049556 | 0.291717044 | 6.829 | 6.618 | 6.3873 | 7.1352 | 8.329 | 8.7736 | 8.4566 |
| CBX3P2 | 0.044994 | 0.27660743 | 1.5267 | 0.78578 | 1.0719 | 1.4638 | 3.0658 | 3.1587 | 2.9739 |
| IL24 | 0.046159 | 0.26969713 | 0.6437 | 0.15348 | 0.21946 | 0.085275 | 2.5358 | 1.9088 | 2.0536 |
| SNIP1 | 0.04934 | 0.251951271 | 2.1919 | 2.7659 | 2.583 | 3.0222 | 4.462 | 4.937 | 4.4896 |
| C20orf111 | 0.032548 | 0.228054414 | 5.0264 | 4.859 | 4.8865 | 5.5291 | 7.0882 | 7.3755 | 7.1597 |
| FBXO33 | 0.005069 | 0.224556439 | 3.8099 | 3.6086 | 3.524 | 3.8117 | 5.8499 | 5.7518 | 5.9285 |
| RPL13AP20 | 0.04998 | 0.18353474 | 1.2005 | 2.0999 | 1.8531 | 2.069 | 4.4746 | 3.8381 | 4.4418 |
| SLC2A14 | 0.048651 | 0.180263524 | 1.5099 | 0.78954 | 0.98851 | 1.0469 | 3.7652 | 2.9956 | 3.9058 |
| ZNF331 | 0.046159 | 0.175216236 | 3.5661 | 2.9224 | 3.341 | 4.0376 | 5.7658 | 6.1615 | 6.0114 |
| ZNF295 | 0.036365 | 0.166622894 | 1.1192 | 1.3023 | 1.5392 | 2.0178 | 4.0134 | 4.3471 | 3.8794 |
| PELI1 | 0.03403 | 0.158504067 | 5.2267 | 5.1173 | 4.4983 | 5.2034 | 7.3275 | 7.9645 | 7.7145 |
| PIGA | 0.036365 | 0.152822984 | 2.1606 | 2.0444 | 2.6286 | 2.8348 | 5.2589 | 5.358 | 4.7646 |
| H3F3C | 0.041834 | 0.150006835 | 2.6202 | 1.9307 | 2.4121 | 1.6154 | 4.7937 | 5.1789 | 4.6719 |
| CABP4 | 0.024142 | 0.125236337 | -1.6186 | -1.4953 | -1.4355 | -2.2421 | 1.2524 | 1.5143 | 1.1315 |
| MECOM | 0.03403 | 0.118796734 | -6.6439 | -6.6439 | -6.6439 | -6.6439 | -4.275 | -3.2259 | -3.2105 |
| B3GNT7 | 0.016021 | 0.100537113 | -6.6439 | -6.6439 | -6.6439 | -6.6439 | -3.6339 | -2.7872 | -3.568 |
| PODNL1 | 0.013123 | 0.083288829 | -6.6439 | -6.6439 | -6.6439 | -6.6439 | -3.3625 | -2.5163 | -3.2957 |
| ABI3BP | 0.04807 | 0.079093338 | -6.6439 | -6.6439 | -6.6439 | -6.6439 | -2.9543 | -2.1073 | -3.8892 |
| ProSAPiP1 | 0.011542 | 0.078685964 | -1.2188 | -1.9039 | -1.5226 | -1.6517 | 2.1798 | 2.3245 | 1.7762 |
| KEL | 0.011486 | 0.068737133 | -6.6439 | -6.6439 | -6.6439 | -6.6439 | -3.0856 | -2.2399 | -3.0179 |
| CLDN1 | 0.032989 | 0.066169041 | -6.6439 | -6.6439 | -6.6439 | -6.6439 | -1.9738 | -2.7122 | -3.4926 |
| SCIN | 0.013717 | 0.055119259 | -6.6439 | -6.6439 | -6.6439 | -6.6439 | -3.1004 | -2.2547 | -2.0327 |
| TEX26-AS1 | 0.008315 | 0.054400606 | -6.6439 | -6.6439 | -6.6439 | -6.6439 | -2.7484 | -1.9035 | -2.6791 |
| GAS2 | 0.008315 | 0.053316776 | -6.6439 | -6.6439 | -6.6439 | -6.6439 | -2.7194 | -1.8746 | -2.6499 |
| LOC339807 | 0.006975 | 0.04584581 | -6.6439 | -6.6439 | -6.6439 | -6.6439 | -2.5018 | -1.6576 | -2.4311 |
| CTAGE11P | 6.11E-05 | 0.044825603 | -6.6439 | -6.6439 | -6.6439 | -6.6439 | -2.1355 | -2.2897 | -2.0679 |
| MTUS2 | 0.020964 | 0.038597029 | -6.6439 | -6.6439 | -6.6439 | -6.6439 | -2.781 | -1.3492 | -1.7154 |
| LOC284648 | 0.006639 | 0.037557205 | -6.6439 | -6.6439 | -6.6439 | -6.6439 | -2.2144 | -1.3712 | -2.1418 |
| TPTE2 | 0.006975 | 0.037207398 | -6.6439 | -6.6439 | -6.6439 | -6.6439 | -2.4655 | -1.6214 | -1.6 |
| GALNT9 | 0.037858 | 0.035359221 | -6.6439 | -6.6439 | -6.6439 | -6.6439 | -2.4605 | -0.61639 | -2.3895 |
| LOC728640 | 0.031017 | 0.022364695 | -6.6439 | -6.6439 | -6.6439 | -6.6439 | -1.0682 | -2.2226 | -0.193 |
| DCT | 0.029118 | 0.02225434 | -6.6439 | -6.6439 | -6.6439 | -6.6439 | -0.11539 | -2.0777 | -1.2693 |
| ETV4 | 0.017416 | 0.020885857 | -6.6439 | -6.6439 | -6.6439 | -6.6439 | -1.8955 | -1.0503 | -0.24191 |
| MATN1 | 0.02132 | 0.020609743 | -6.6439 | -6.6439 | -6.6439 | -6.6439 | -2.0749 | -0.49968 | -0.55553 |
| SYT4 | 0.03403 | 0.019251753 | -6.6439 | -6.6439 | -6.6439 | -6.6439 | -2.2356 | -0.16631 | -0.43319 |
| EBF4 | 0.048626 | 0.019103798 | -6.6439 | -6.6439 | -6.6439 | -6.6439 | -0.70994 | -2.4488 | 0.35703 |
| CLDND2 | 0.004712 | 0.01772857 | -6.6439 | -6.6439 | -6.6439 | -6.6439 | -0.46591 | -0.62645 | -1.386 |
| ZNF876P | 0.006975 | 0.016363256 | -6.6439 | -6.6439 | -6.6439 | -6.6439 | -0.80035 | -1.2765 | -0.05466 |
| MGC14436 | 0.002183 | 0.014663772 | -6.6439 | -6.6439 | -6.6439 | -6.6439 | -0.13491 | -0.8791 | -0.64289 |
| UPK3A | 0.004712 | 0.013198095 | -6.6439 | -6.6439 | -6.6439 | -6.6439 | -0.76807 | 0.24378 | -0.67683 |
| ANP32AP1 | 0.004712 | 0.012202786 | -6.6439 | -6.6439 | -6.6439 | -6.6439 | -0.92841 | -0.0931 | 0.15975 |
| GNG3 | 0.017416 | 0.011918862 | -6.6439 | -6.6439 | -6.6439 | -6.6439 | 0.16346 | 0.41767 | -1.341 |
| TPD52L1 | 2.08E-05 | 0.009786702 | -6.6439 | -6.6439 | -6.6439 | -6.6439 | 0.058787 | -0.09926 | 0.13366 |
| LOC100303749 | 0.024652 | 0.009525917 | -6.6439 | -6.6439 | -6.6439 | -6.6439 | 1.3104 | -0.17547 | -0.92485 |
| PPIAL4F | 0.004712 | 0.008380533 | -6.6439 | -6.6439 | -6.6439 | -6.6439 | 0.94738 | -0.21621 | 0.033356 |
| UBE2NL | 0.013812 | 0.00785872 | -6.6439 | -6.6439 | -6.6439 | -6.6439 | 0.76506 | 1.0216 | -0.74389 |
| TNNC2 | 0.013795 | 0.003717007 | -6.6439 | -6.6439 | -6.6439 | -6.6439 | 0.23813 | 2.3937 | 1.6514 |
