# Supplementary material for: Transcriptional profiling demonstrates altered characteristics of CD8 + cytotoxic T‐cells and regulatory T‐cells in TP53‐mutated acute myeloid leukemia
Source: Cancer Med. 2022 Mar 16;11(15):3023–32. doi: 10.1002/cam4.4661 (PMC9359873; doi:10.1002/cam4.4661)
Supplement: Supplementary file 5 — TableS 4 [file CAM4-11-3023-s004.docx]

**Supporting table 4**

Differentially expressed genes by AML Th vs. control Th.

| **Gene name** | **FDR<0.05** | **Fold change** | **Expression levels (log2 FPKM values)** | | | | | |
| --- | --- | --- | --- | --- | --- | --- | --- | --- |
|  |  |  | **AML-A** | **AML-C** | **AML-D** | **Normal-A** | **Normal-B** | **Normal-C** |
| WBP5 | 0.030257 | 439.7071928 | 2.5287 | 1.1474 | 2.7334 | -6.6439 | -6.6439 | -6.6439 |
| HIST1H4B | 0.011771 | 302.4947986 | 1.9608 | 1.0158 | 1.814 | -6.6439 | -6.6439 | -6.6439 |
| HLA-DQB1 | 0.03698 | 271.3560949 | 0.39943 | 2.0368 | 1.8842 | -6.6439 | -6.6439 | -6.6439 |
| ARL17B | 0.041137 | 194.0637196 | 1.9206 | 0.10199 | 0.84687 | -6.6439 | -6.6439 | -6.6439 |
| HMOX1 | 0.041137 | 190.7082501 | -0.034129 | 1.7665 | 1.0616 | -6.6439 | -6.6439 | -6.6439 |
| LOC100286938 | 0.012118 | 150.6023078 | 0.28481 | 0.36589 | 1.1214 | -6.6439 | -6.6439 | -6.6439 |
| ANG | 0.002163 | 147.1842932 | 0.61235 | 0.71392 | 0.34647 | -6.6439 | -6.6439 | -6.6439 |
| LOC100507173 | 0.039585 | 90.3478823 | -0.98762 | 0.47281 | 0.075368 | -6.6439 | -6.6439 | -6.6439 |
| LOC440131 | 0.025378 | 88.49339205 | -0.82319 | 0.28425 | 0.009734 | -6.6439 | -6.6439 | -6.6439 |
| LILRB1 | 0.039585 | 88.4871623 | -0.14081 | 0.55604 | -0.94474 | -6.6439 | -6.6439 | -6.6439 |
| SLC16A4 | 0.002163 | 87.30877717 | -0.23926 | -0.34807 | -0.00021 | -6.6439 | -6.6439 | -6.6439 |
| DUSP19 | 0.036916 | 82.58147908 | -1.0546 | 0.29615 | -0.07001 | -6.6439 | -6.6439 | -6.6439 |
| CLIC4 | 0.039585 | 81.38546404 | 0.020157 | 0.23554 | -1.1473 | -6.6439 | -6.6439 | -6.6439 |
| RAD51AP1 | 0.039585 | 76.83683743 | 0.38377 | -0.45489 | -1.0694 | -6.6439 | -6.6439 | -6.6439 |
| LOC100287177 | 0.030257 | 59.77706888 | -1.3916 | -0.27671 | -0.55883 | -6.6439 | -6.6439 | -6.6439 |
| SMA4 | 0.02535 | 58.31762192 | -0.6024 | -0.36922 | -1.3625 | -6.6439 | -6.6439 | -6.6439 |
| B3GNT3 | 0.036916 | 55.50128733 | -0.22567 | -1.5146 | -0.80808 | -6.6439 | -6.6439 | -6.6439 |
| LOC100289656 | 0.020647 | 49.48674155 | -1.3306 | -1.2164 | -0.49779 | -6.6439 | -6.6439 | -6.6439 |
| TMEM44 | 0.006727 | 44.99772883 | -1.4071 | -0.86816 | -1.1811 | -6.6439 | -6.6439 | -6.6439 |
| CEBPE | 0.002163 | 39.9179683 | -1.3099 | -1.1877 | -1.4772 | -6.6439 | -6.6439 | -6.6439 |
| CIDEC | 0.02535 | 34.11576579 | -1.8689 | -1.7496 | -1.0361 | -6.6439 | -6.6439 | -6.6439 |
| CCDC13 | 0.002163 | 33.48941563 | -1.7029 | -1.5766 | -1.4553 | -6.6439 | -6.6439 | -6.6439 |
| SLCO4C1 | 0.007155 | 32.70033047 | -1.6738 | -1.323 | -1.8412 | -6.6439 | -6.6439 | -6.6439 |
| KIF15 | 0.041137 | 30.04527456 | -1.5268 | -2.3986 | -1.2791 | -6.6439 | -6.6439 | -6.6439 |
| APOF | 0.041137 | 28.6176506 | -2.457 | -1.3339 | -1.6243 | -6.6439 | -6.6439 | -6.6439 |
| PIP5K1B | 0.030257 | 28.50415481 | -1.602 | -1.476 | -2.3544 | -6.6439 | -6.6439 | -6.6439 |
| UGT8 | 0.031258 | 25.86687193 | -1.7422 | -1.6158 | -2.4946 | -6.6439 | -6.6439 | -6.6439 |
| CD19 | 0.031258 | 24.21283524 | -1.6989 | -2.5747 | -1.865 | -6.6439 | -6.6439 | -6.6439 |
| WNT2B | 0.047208 | 23.3761681 | -2.7493 | -1.6249 | -1.9166 | -6.6439 | -6.6439 | -6.6439 |
| GRPR | 0.002212 | 21.51344204 | -2.203 | -2.0769 | -2.3703 | -6.6439 | -6.6439 | -6.6439 |
| RARB | 0.002212 | 20.72882776 | -2.2566 | -2.1304 | -2.424 | -6.6439 | -6.6439 | -6.6439 |
| SEMA3G | 0.002163 | 19.50627317 | -2.4833 | -2.3552 | -2.2356 | -6.6439 | -6.6439 | -6.6439 |
| CALHM2 | 0.034281 | 6.445832286 | 4.6408 | 4.4477 | 4.5741 | 1.7232 | 1.7026 | 2.1717 |
| C14orf119 | 0.039585 | 6.049293714 | 5.3537 | 5.16 | 5.2638 | 2.7544 | 2.89 | 2.3428 |
| HEMGN | 0.036916 | 5.624242061 | 2.3917 | 2.5166 | 2.0639 | -0.12399 | -0.04516 | -0.33363 |
| LPAR6 | 0.02535 | 5.273398926 | 6.9069 | 6.7056 | 6.8294 | 4.5499 | 4.4927 | 4.2031 |
| ARV1 | 0.023415 | 5.012974531 | 4.6002 | 4.3703 | 4.2235 | 2.1174 | 2.0712 | 2.0284 |
| RAB10 | 0.043426 | 4.379298753 | 6.0787 | 5.9628 | 5.6867 | 3.6992 | 3.6621 | 3.9748 |
| CETN3 | 0.041698 | 3.904848848 | 4.5178 | 4.4942 | 4.7877 | 2.4349 | 2.752 | 2.717 |
| CDK2AP2 | 0.023415 | 3.701303987 | 6.191 | 6.4759 | 6.3853 | 4.5199 | 4.3928 | 4.4754 |
| THAP2 | 0.039585 | 3.493457121 | 1.8415 | 1.6302 | 1.9679 | 0.040654 | -0.12617 | 0.11115 |
| BET1 | 0.036916 | 3.098421173 | 3.6664 | 3.6111 | 3.651 | 2.0622 | 2.1586 | 1.8131 |
| HMGCL | 0.049407 | 2.838706171 | 4.205 | 4.2396 | 4.3646 | 2.9585 | 2.6026 | 2.7324 |
| SDF2 | 0.043426 | 2.7896803 | 5.6171 | 5.482 | 5.3417 | 3.986 | 4.1269 | 3.8876 |
| AP1S2 | 0.035897 | 2.42721175 | 4.9589 | 4.9051 | 4.7301 | 3.5577 | 3.5362 | 3.6623 |
| ICMT | 0.036916 | 2.257094167 | 2.8839 | 3.012 | 3.1028 | 1.7489 | 1.8816 | 1.8448 |
| CLNS1A | 0.036916 | 2.249181516 | 6.1556 | 6.0589 | 6.03 | 4.9649 | 4.786 | 4.9854 |
| TPST2 | 0.020647 | 2.139341471 | 5.2121 | 5.2271 | 5.2635 | 4.1147 | 4.23 | 4.0665 |
| ICAM2 | 0.013278 | 2.075750912 | 7.6308 | 7.5458 | 7.6054 | 6.5684 | 6.5759 | 6.4768 |
| NEK4 | 0.030257 | 2.056085223 | 2.9582 | 2.8055 | 2.9513 | 1.8972 | 1.8016 | 1.8965 |
| FANCF | 0.044273 | 1.949133931 | 3.1966 | 3.3901 | 3.3237 | 2.3204 | 2.2834 | 2.4181 |
| MRPL12 | 0.047686 | 1.804459386 | 4.29 | 4.3817 | 4.3523 | 3.4721 | 3.4001 | 3.5971 |
| PARP1 | 0.024829 | 1.732073269 | 5.6626 | 5.5796 | 5.5891 | 4.7615 | 4.8314 | 4.8609 |
| RRAGA | 0.030257 | 1.421059135 | 5.8059 | 5.7655 | 5.8155 | 5.2494 | 5.3324 | 5.2842 |
| MAP9 | 0.031258 | 1.404328116 | 1.1474 | 1.2266 | 1.1848 | 0.66747 | 0.72652 | 0.69517 |
| NENF | 0.020647 | 0.773675151 | 5.1867 | 5.1933 | 5.2073 | 5.5952 | 5.5398 | 5.5629 |
| NACA | 0.041137 | 0.742656394 | 8.3984 | 8.3765 | 8.3654 | 8.7663 | 8.8001 | 8.8616 |
| HMGB1 | 0.047686 | 0.73579037 | 7.2368 | 7.1769 | 7.1287 | 7.6379 | 7.6307 | 7.6017 |
| RSL1D1 | 0.030257 | 0.730336578 | 5.2858 | 5.3035 | 5.3585 | 5.7749 | 5.744 | 5.789 |
| PLEKHA1 | 0.004085 | 0.681443806 | 4.8468 | 4.8051 | 4.8364 | 5.386 | 5.3746 | 5.3877 |
| NFIC | 0.032891 | 0.593299801 | 1.2628 | 1.3173 | 1.2062 | 2.0668 | 1.9578 | 2.0212 |
| ATP5S | 0.004814 | 0.582878288 | 2.613 | 2.6501 | 2.5903 | 3.3807 | 3.3959 | 3.413 |
| ECHDC2 | 0.035897 | 0.571014792 | 4.1439 | 4.0919 | 4.2366 | 5.0147 | 4.9231 | 4.9598 |
| PABPC1 | 0.043426 | 0.556993775 | 7.9453 | 8.087 | 7.9173 | 8.7676 | 8.8613 | 8.8535 |
| QRICH1 | 0.049407 | 0.553274311 | 5.0693 | 5.0904 | 5.122 | 5.9933 | 6.0274 | 5.8228 |
| VAMP2 | 0.006727 | 0.545795821 | 5.8311 | 5.8317 | 5.866 | 6.6714 | 6.7339 | 6.7442 |
| RLIM | 0.018815 | 0.519921804 | 2.281 | 2.2192 | 2.1853 | 3.21 | 3.1932 | 3.1132 |
| SMCR8 | 0.035975 | 0.518158933 | 1.8369 | 1.8224 | 1.666 | 2.6931 | 2.7671 | 2.7107 |
| POLR3E | 0.047208 | 0.514282626 | 3.2686 | 3.3082 | 3.4824 | 4.3493 | 4.3278 | 4.2602 |
| INSIG1 | 0.039585 | 0.494314195 | 4.5014 | 4.3888 | 4.609 | 5.5333 | 5.542 | 5.4734 |
| PITPNC1 | 0.028386 | 0.493686276 | 5.5236 | 5.5285 | 5.6846 | 6.5913 | 6.5795 | 6.6209 |
| 06-Mar | 0.003788 | 0.483772266 | 4.0693 | 4.0944 | 4.1243 | 5.1362 | 5.117 | 5.1776 |
| FOXN3 | 0.006727 | 0.479521241 | 3.8157 | 3.8425 | 3.9155 | 4.9281 | 4.9244 | 4.9022 |
| RBBP6 | 0.031258 | 0.466300977 | 4.904 | 4.9116 | 4.7965 | 5.9313 | 6.0748 | 5.908 |
| NECAP1 | 0.017567 | 0.458247878 | 5.0119 | 4.8715 | 4.8762 | 6.0644 | 6.0316 | 6.041 |
| TOMM20 | 0.020647 | 0.454200087 | 5.1951 | 5.226 | 5.3589 | 6.4273 | 6.3981 | 6.3704 |
| ZNF326 | 0.011771 | 0.425117418 | 2.9673 | 2.9976 | 3.0448 | 4.1911 | 4.3136 | 4.2072 |
| ZNF136 | 0.048507 | 0.401647003 | 4.0039 | 3.6924 | 3.92 | 5.1198 | 5.233 | 5.2115 |
| ELF2 | 0.002212 | 0.40075706 | 4.4823 | 4.4412 | 4.4838 | 5.8144 | 5.8004 | 5.7501 |
| OTUD1 | 0.039585 | 0.382261876 | 3.9471 | 4.091 | 4.1509 | 5.3067 | 5.5274 | 5.517 |
| CBX4 | 0.047686 | 0.275896701 | 3.2754 | 3.6002 | 3.4815 | 5.4969 | 5.2873 | 5.1463 |
| FBXO33 | 0.042584 | 0.246142717 | 4.0746 | 3.7423 | 3.9393 | 6.0349 | 6.0577 | 5.7309 |
| JMY | 0.047208 | 0.240993608 | 2.1682 | 2.198 | 2.5793 | 4.3474 | 4.506 | 4.2509 |
| FAM83D | 0.030257 | 0.226209286 | -0.23948 | -0.33632 | -0.21421 | 1.6869 | 2.0875 | 1.8684 |
| BMPER | 0.030257 | 0.101255126 | -6.6439 | -6.6439 | -6.6439 | -3.0177 | -3.3544 | -3.6478 |
| HEATR4 | 0.017783 | 0.043150864 | -6.6439 | -6.6439 | -6.6439 | -2.4547 | -1.7909 | -2.0827 |
| GABARAPL3 | 0.012142 | 0.035113439 | -6.6439 | -6.6439 | -6.6439 | -1.4923 | -1.8284 | -2.1155 |
| C19orf45 | 0.011771 | 0.030782767 | -6.6439 | -6.6439 | -6.6439 | -1.3029 | -1.6389 | -1.9247 |
| FAM66D | 0.007155 | 0.017999785 | -6.6439 | -6.6439 | -6.6439 | -0.53184 | -0.86703 | -1.1452 |
| C15orf48 | 0.002212 | 0.013905827 | -6.6439 | -6.6439 | -6.6439 | -0.24738 | -0.58216 | -0.59766 |
| ADRA2B | 0.020647 | 0.01382014 | -6.6439 | -6.6439 | -6.6439 | -0.03799 | -0.37455 | -0.98791 |
| CRABP2 | 0.032456 | 0.010973863 | -6.6439 | -6.6439 | -6.6439 | 0.51534 | -0.82035 | -0.09734 |
